# Heterozygous *Dab1* Null Mutation Disrupts Neocortical and Hippocampal Development

**DOI:** 10.1523/ENEURO.0433-22.2023

**Published:** 2023-04-07

**Authors:** Takao Honda, Yuki Hirota, Kazunori Nakajima

**Affiliations:** 1 Department of Anatomy, Keio University School of Medicine, Tokyo 160-8582, Japan; 2 Laboratory of Molecular Biology, Department of Biofunctional Analysis, Gifu Pharmaceutical University, Gifu 501-1196, Japan

**Keywords:** apical dendrite, Dab1, haploinsufficiency, *reeler*, Reelin, *yotari*

## Abstract

Loss-of-function mutations in Reelin and DAB1 signaling pathways disrupt proper neuronal positioning in the cerebral neocortex and hippocampus, but the underlying molecular mechanisms remain elusive. Here, we report that heterozygous *yotari* mice harboring a single autosomal recessive *yotari* mutation of *Dab1* exhibited a thinner neocortical layer 1 than wild-type mice on postnatal day (P)7. However, a birth-dating study suggested that this reduction was not caused by failure of neuronal migration. *In utero* electroporation-mediated sparse labeling revealed that the superficial layer neurons of heterozygous *yotari* mice tended to elongate their apical dendrites within layer 2 than within layer 1. In addition, the CA1 pyramidal cell layer in the caudo-dorsal hippocampus was abnormally split in heterozygous *yotari* mice, and a birth-dating study revealed that this splitting was caused mainly by migration failure of late-born pyramidal neurons. Adeno-associated virus (AAV)-mediated sparse labeling further showed that many pyramidal cells within the split cell had misoriented apical dendrites. These results suggest that regulation of neuronal migration and positioning by Reelin-DAB1 signaling pathways has unique dependencies on *Dab1* gene dosage in different brain regions.

## Significance Statement

DAB1 is a cytoplasmic adaptor protein essential for transmission of the extracellular Reelin signal to cytoplasmic proteins that regulate cortical development. In this study, we found that *Dab1* is haplosufficient for the regulation of neuronal migration but haploinsufficient for control of layer 1 thickness in the cerebral neocortex. Alternatively, the migration of a subpopulation of hippocampal pyramidal neurons is sensitive to *Dab1* gene haploinsufficiency. This study suggests that neural development in the cerebral neocortex and hippocampus are differentially sensitive to *Dab1* gene dose.

## Introduction

In the mammalian cerebral neocortex, excitatory neurons are born mainly in the ventricular and subventricular zones and migrate radially toward the pial surface by passing through preexisting neuronal layers. Therefore, excitatory cortical neurons are aligned in an “early-born deep” and “late-born superficial” sequence (Cooper, 2008; Honda et al., 2011; Ohtaka-Maruyama and Okado, 2015). Radially migrating neurons express receptors for Reelin, a large secreted glycoprotein mainly produced by Cajal-Retzius cells in the marginal zone that is critical for the positioning of excitatory neurons (D’Arcangelo et al., 1995; Hirotsune et al., 1995; Ogawa et al., 1995). Activation of the Reelin receptors apolipoprotein E receptor 2 (APOER2) and/or very low-density lipoprotein receptor (VLDLR) stimulates Src-mediated tyrosine phosphorylation of the nucleocytoplasmic-shuttling adaptor protein DAB1 (Howell et al., 1997, 2000; Trommsdorff et al., 1999; Honda and Nakajima, 2006, 2016), which in turn interacts with a host of molecular pathways controlling neuronal migration (Assadi et al., 2003; Bock et al., 2003; Pramatarova et al., 2003; Ballif et al., 2004; Chen et al., 2004; Huang et al., 2004; Feng et al., 2007; Franco et al., 2011; Jossin and Cooper, 2011; Sekine et al., 2012; Matsunaga et al., 2017). Thus, the null mutation of *Reelin* (Falconer, 1951; Montgomery et al., 1994; Bar et al., 1995; D’Arcangelo et al., 1995), the double null mutation of *Apoer2* and *Vldlr* (D’Arcangelo et al., 1999; Hiesberger et al., 1999; Trommsdorff et al., 1999), and *Dab1* null mutation (Howell et al., 1997; Sheldon et al., 1997; Yoneshima et al., 1997; Kojima et al., 2000) result in developmental defects in the cerebral neocortex, hippocampus, and cerebellum. In cerebral neocortex, for instance, *Reelin*, or *Dab1* null mutation results in the absence of layer 1 and severe neuronal migration failure (Hamburgh, 1963; Caviness, 1982; D’Arcangelo, 2005). Similarly, excessive cytosolic DAB1 protein causes severe migration failure in wild-type mice (Honda and Nakajima, 2016), suggesting that the regulation of cytosolic DAB1 protein amount is important for the migration of excitatory neurons. Naturally occurring deletions, duplications, and loss-of-function mutations of genes sometimes cause gene dosage effects on function (Veitia and Potier, 2015). For diploid organisms, the protein product from one allele is often sufficient to allow normal growth, development, and physiological function; however, a small number of genes exhibit dosage sensitivity, leading to deficits in the heterozygous state (termed haploinsufficiency). In general, genes encoding enzymes tend to be dose-insensitive, whereas genes that encode proteins with structural, transcriptional, or regulatory functions are more likely to be dose-sensitive (Kondrashov and Koonin, 2004; Morrill and Amon, 2019). DAB1 is a versatile protein that mediates multiple functions, but the underlying signaling pathways for many of these functions have not been fully elucidated. Therefore, it is important to dissect DAB1 pathways and determine the gene-dose sensitivity of each. Further, among the previous studies related to the Reelin-DAB1 signaling pathway, there are several reports showing that attenuation of Reelin signaling pathway caused defects in the cerebral cortical development (Herrick and Cooper, 2002; Hack et al., 2007; Kohno et al., 2015; de Frutos et al., 2016; Hirota et al., 2018; Hirota and Nakajima, 2020) and might be associated with the pathogenesis of a number of neuropsychiatric disorders including schizophrenia (Impagnatiello et al., 1998; Guidotti et al., 2000), bipolar disorder (Guidotti et al., 2000), autism spectrum disorder (Fatemi et al., 2001, 2005), and Alzheimer’s disease (Chin et al., 2007). However, it is not clear whether there is *Dab1* gene-dosage dependency for each reported phenotype.

To examine whether *Dab1* exhibits haploinsufficiency or haplosufficiency in various neurodevelopmental functions, we compared neocortical and hippocampal structures between wild-type and heterozygous *yotari* mice harboring a single *Dab1* null mutation (Sheldon et al., 1997; Yoneshima et al., 1997; Kojima et al., 2000). We found that heterozygous *yotari* mice at postnatal day (P)7 exhibited reduced neocortical layer 1 thickness. In addition, some neurons located in the superficial layer tended to elongate their dendrites ectopically within layer 2. Also, we found that the late-born CA1 pyramidal cell layer in the caudal hippocampus is abnormally split in heterozygous *yotari* mice. These findings suggest that the *Dab1* gene dosage is important for maintenance of the layer 1 thickness and dendrite guidance of superficial-layer neurons in the cerebral neocortex and for the migration of late-born pyramidal neurons in the caudal hippocampus.

## Materials and Methods

### Mice

Heterozygous *yotari* or wild-type females were mated with heterozygous *yotari* males (RRID:IMSR_RBRC05456). Noon on the day the vaginal plug was found was designated as embryonic day (E)0.5. All animal procedures were performed in accordance with the Keio University Animal Care and Use Committee’s regulations, Japanese Government Law Concerning the Protection and Control of Animals, and Japanese Government Notification of Feeding and Safekeeping of Animals.

### Plasmids

A fluorescent protein expression vector, pCAGGS-RG, capable of Cre-dependent switching from DsRed to enhanced green fluorescent protein (EGFP) expression, and a Cre expression vector, pDCX-Cre, expressing Cre under control of the *Doublecortin* (DCX) promoter were used for sparse labeling of neurons as has been shown before (Kohno et al., 2015). To construct an adeno-associated virus (AAV)-DCX-Cre vector, the DCX promoter was obtained from Dcx4kb-EGFP (X. Wang et al., 2007; kindly provided by Q. Lu, Beckman Research Institute of the City of Hope), Cre cDNA was obtained from pCXN-Cre (Koresawa et al., 2000; kindly provided by S. Miyagawa, Osaka University), and the AAV plasmid backbone were obtained from AAV-CAG-GFP (a kind gift from K. Svoboda, Addgene plasmid #28014; http://n2t.net/addgene:28014; RRID:Addgene_28014). To construct AAV-CAG-Double-floxed Inverse Orientation (DIO)-EGFP and AAV-CAG-DIO-mCherry vectors, the DIO cassette sequence information was obtained from the double-floxed inverse ChR2-EYFP vector (Sohal et al., 2009), and the sequence was synthesized by Genscript. This DIO cassette was then inserted into the AAV-CAG backbone obtained from AAV-CAG-GFP, yielding AAV-CAG-DIO1. The cDNAs encoding EGFP and mCherry were amplified by PCR from pEGFP-N1 (Clontech) and pmCherry (Clontech), respectively, and inserted into AAV-CAG-DIO1 in reverse orientation with respect to the CAG promoter to allow Cre-dependent expression of the reporter genes. The adenovirus helper plasmid pAdΔF6 and vector pAAV2/9 were kindly provided by the University of Pennsylvania Penn Vector Core.

### Bromodeoxyuridine (BrdU) labeling

Bromodeoxyuridine (Merck) was first dissolved in sterile phosphate-buffered saline (PBS) at 10 mg/ml, and the solution was administered to pregnant dams by intraperitoneal injection at 50 μg BrdU/g body weight on gestation days 12.5, 14.5, and 16.5.

### Immunohistochemistry

Mouse pups treated as indicated were anesthetized using isoflurane and brains fixed by transcardial perfusion with 4% paraformaldehyde (PFA). Brains were then isolated, postfixed in PFA at 4°C for 2 h, washed with PBS, incubated sequentially in 10%, 20%, and 30% sucrose/PBS for cryopreservation, embedded in a mixture of optimal cutting temperature (OCT) compound (Sakura Finetek) and 30% sucrose/PBS solution, and cryosectioned in the coronal plane at 20 μm thick. For layer marker staining, sections were washed three times (5 min/wash) in PBS containing 0.1% Tween 20 (PBStw) at room temperature (RT), autoclaved at 105°C for 5 min in 0.01 m citrate buffer (pH 6.0) for antigen retrieval, then incubated sequentially with 5% bovine serum albumin (BSA)/PBStw at RT for 5 min and primary antibodies against BRN2 (Santa Cruz Biotechnology, sc-6029; RRID:AB_2167385), RORB (Perseus Proteomics, PP-N7927-00; RRID:AB_1964364), or TBR1 (abcam, ab31940; RRID:AB_2200219) in 5% BSA/PBStw at RT for 1 h. For visualization of immunolabeling, sections were washed three times in PBStw and incubated first with Alexa 488-labeled secondary antibody against mouse IgG (Thermo Fisher Scientific, A-21202; RRID:AB_141607) or rabbit mouse IgG (Thermo Fisher Scientific, A-11008; RRID:AB_143165) with 4′,6-diamidino-2-phenylindole (DAPI; Life Technologies) at RT for 1 h. After three washes in PBS, sections were mounted with PermaFluor Aqueous Mounting Medium (Thermo Fisher Scientific). For double staining with BrdU and either BRN2 or CTIP2 (official symbol is BCL11B) antibodies, sections were autoclaved, blocked with 5% BSA/PBStw, incubated with goat anti-BRN2 (Santa Cruz, sc-6029; RRID:AB_2167385) or rat anti-CTIP2 (abcam, ab18465; RRID:AB_2064130) in 5% BSA/PBStw, washed, and incubated with Alexa 555-labeled anti-goat IgG (Thermo Fisher Scientific, A-21432; RRID:AB_2535853) or Alexa 594-labeled anti-rat IgG (Thermo Fisher Scientific, A-21209; RRID:AB_2535795). Sections were rinsed with PBStw, fixed in 4% PFA for 10 min, washed three times in PBStw (5 min/wash) to remove trace PFA, incubated with 2N HCl at 37°C for 30 min, rewashed three times in PBStw (5 min/wash) to remove HCl, blocked in 5% BSA/PBStw for 5 min, incubated with mouse anti-BrdU (BD Biosciences, 347580; RRID:AB_10015219) for 1 h, washed again three times in PBStw, and incubated with an Alexa 488-labeled secondary antibody against mouse IgG (Thermo Fisher Scientific, A-21202; RRID:AB_141607) containing DAPI for 1 h. After three washes in PBStw, sections were embedded in PermaFluor. For DAPI staining alone, cryosections were washed in PBStw for 5 min, incubated with PBStw containing DAPI for 5 min, washed with PBStw, and embedded in PermaFluor. For immunohistochemical staining of mCherry in the hippocampus, OCT-embedded brains were sectioned in the coronal plane at 100 μm thick and floated on PBS in a 12-well plates. Selected sections were transferred to 24-well plates containing PBS, and treated with PBS containing 5% BSA and 0.5% Triton X-100 (blocking/permeabilization buffer) for 30 min to 1 h at RT on a shaker. After removal of the blocking/permeabilization buffer, sections were treated with primary antibody against RFP (Rockland, 600-401-379; RRID:AB_2209751) at 4°C overnight on a shaker, washed three times with blocking/permeabilization buffer, and incubated with Alexa Fluor 555-labeled secondary antibody against rabbit IgG (Thermo Fisher Scientific, A-31 572; RRID:AB_162543) containing DAPI for 2–3 h on a shaker. After three additional washes in PBS, the sections were embedded in PermaFluor. Confocal images were acquired using an Olympus FV1000 confocal microscope (Olympus), Leica SP8 confocal microscope (Leica microsystems), or Zeiss LSM700 confocal microscope (Zeiss).

### Nissl staining

For Nissl staining, OCT-embedded brains obtained from P0 or P7 pups were sectioned in the coronal plane at 20 μm thick, and the sections floated on a small droplet of distilled water (DW) placed on a glass slide. The excess DW under the section was removed with a pipette and allowed to air dry. Then, sections were washed three times with PBS (5 min/wash), dehydrated in an ascending ethanol gradient (70%, 80%, 90%, 95%, 99%, and 100%, each for 1 min), and then rehydrated in a descending ethanol gradient (100%, 99%, 95%, 90%, 80%, and 70%, each for 1 min). Sections were treated with 0.1% cresyl violet solution for 30 min, washed with DW for 5 min, destained with 0.005% acetic acid containing absolute ethanol for 30 s, and washed with DW for 5 min. Stained sections were dehydrated in the same ascending ethanol gradient, cleared with 100% xylene, and mounted with Entellan mounting medium (Merck).

### *In utero* electroporation

*In utero* electroporation was conducted as previously described (Tabata and Nakajima, 2001, 2008). We first prepared an anesthetic mixture of three drugs in PBS, 75 ng/ml medetomidine hydrochloride (Domitor; Nippon Zenyaku Kogyo), 400 ng/ml midazolam (Sandoz), and 0.5 mg/ml butorphanol (Vetorphale; Meiji Seika Pharma). Pregnant mice at E16.5 were deeply anesthetized by the three-drug mixture at 10 μl/body weight (g), and the uterus was exteriorized. Plasmid DNA solution containing 2.5 μg/μl pCAGGS-RG and 200 ng/μl pDCX-Cre supplemented with 0.1% fast green at 1:10 v/v was injected into the lateral ventricle of embryos using a glass capillary. Then, while holding the head of individual embryos with a tweezer-type electrode (CUY21; NEPA Gene) from outside the uterine wall, 35-V electric pulses were applied four times, each for 50 ms at 950-ms intervals, using an electroporator (NEPA21; NEPA Gene). The uterus was then pushed back into the abdominal cavity, and 75 ng/ml atipamezole hydrochloride (Antisedan; Nippon Zenyaku kogyo) solution dissolved in PBS was injected at the same volume as the three-drug anesthetic mixture. Electroporated mice allowed to continue gestation and fixed by transcardial perfusion with 4% PFA at P7. Cryosections were prepared as described above.

### Recombinant adeno-associated virus (rAAV) preparation

Recombinant adeno-associated viruses were prepared as described previously (Burger and Nash, 2016). A mixture of plasmid DNAs including (1) AAV-DCX-Cre, pAdΔF6, and pAAV2/9, (2) AAV-CAG-DIO-EGFP, pAdΔF6, and pAAV2/9, or (3) AAV-CAG-DIO-mCherry, pAdΔF6, and pAAV2/9, was used to transfect AAVpro 293T cells (Takara Bio) seeded on twenty 15-cm dishes using polyethyleneimine “Max” (PEI-Max; Polysciences). After 2 d, cells were collected and lysed by three freeze–thaw treatments and lysates treated with Benzonase nuclease (Millipore). The rAAVs were purified from the lysates by ultracentrifugation using a discontinuous gradient of iodixanol (OptiPrep; Abbott Diagnostics Technologies AS). Banded rAAVs were collected from the 40% iodixanol layer, and the iodixanol buffer exchange to PBS and concentration of the rAAV was performed using a Vivaspin 20 ultrafiltration unit (100 kDa molecular weight cut off; Sartorius). For determination of the genomic titer, rAAVs were treated sequentially with DNaseI and Proteinase K, and the quantity of viral DNAs was determined by quantitative PCR using a StepOnePlus real-time PCR system (Applied Biosystems).

### rAAV injection

New-born pups (P0) were anesthetized on ice, and injected with AAV-DCX-Cre at ∼6 × 10^8^ vector genomes (vg)/μl, AAV-CAG-DIO-EGFP at ∼2 × 10^8^ vg/μl, and AAV-CAG-DIO-mCherry at ∼2 × 10^8^ vg/μl supplemented with 1:10 (v/v) 0.1% fast green into the lateral ventricles at 3 μl per ventricle using glass capillary connected to a microinjector. The rAAV-injected pups were placed on a warming plate until they regained normal color and full typical new-born activity, and then returned to their mothers. On P7, rAAV-injected mice were fixed by transcardial perfusion of 4% PFA and 100-μm-thick brain cryosections were prepared in the coronal plane as described above.

### Measurement of layer 1, cortical wall, and relative layer 1 thickness

Layer thicknesses were measured on digital images of brain coronal sections taken from the dorsolateral region of the rostral (somatosensory and auditory), middle (visual, parietal, and auditory), and caudal (visual and auditory) cortex using Fiji software (RRID:SCR_002285; Schindelin et al., 2012). To measure neocortical layer 1 thickness, a straight line was drawn along the shortest radial distance from the pial surface to the ventricular surface (Fig. 2*A*,*H*; Extended Data Fig. 2-1). Intersecting points with the pial surface, layer 1–layer 2 border, and ventricular surface were designated A, B, and C, respectively. First, layer 1 thickness between points A and B, and cortical wall thickness between points A and C were measured. Then, a point A + 1 located 100 μm to the right of point A was drawn on the pial surface. Similarly, points A + 2 to A + 5 were drawn on the pial surface to the right of A + 1, while points A − 1 to A − 5 were drawn on the pial surface to the left of point A. Straight lines were drawn toward the ventricular surface from points A + 1 to A + 5 and A − 1 to A − 5. These lines were measured as layer 1 thickness and cortical wall thickness, respectively, using Fiji software. Measurements were performed using six P7 brains from six different litters. Relative layer 1 thickness was calculated by dividing layer 1 thickness by cortical wall thickness and multiplying by 100. Measurement results were compared between groups by independent samples Student’s *t* test using the R language environment (R Foundation for Statistical Computing; https://www.r-project.org/; RRID:SCR_001905) as described in Table 1.

**Table 1:** Summary of statistical analyses

Figure number	Panel	Comparison	Data structure: normality	Data structure: homoscedasticity	Type of test	95% confidence interval/Z	*p-*value
Fig. 2	*I*	Rostral: wild type vs heterozygote	Normal distribution Shapiro–Wilk normality test wild type: W = 0.92806, *p-*value = 0.5652 heterozygote: W = 0.79679, *p-*value = 0.05498	Equal variance *F* test *p-*value = 0.3117	Unpaired Student’s *t* test	12.43746 to 40.41903	0.002
Fig. 2	*I*	Middle: wild type vs heterozygote	Normal distribution Shapiro–Wilk normality test wild type: W = 0.92585, *p-*value = 0.5484 heterozygote: W = 0.94264, *p-*value = 0.6805	Equal variance *F* test *p-*value = 0.4658	Unpaired Student’s *t* test	10.76698 to 42.59023	0.004
Fig. 2	*I*	Caudal: wild type vs heterozygote	Normal distribution Shapiro–Wilk normality test wild type: W = 0.956, *p-*value = 0.7885 heterozygote: W = 0.90402, *p-*value = 0.3982	Equal variance *F* test *p-*value = 0.6094	Unpaired Student’s *t* test	13.47947 to 41.17302	0.001
Fig. 2	*J*	Rostral: wild type vs heterozygote	Normal distribution Shapiro–Wilk normality test wild type: W = 0.93972, *p-*value = 0.657 heterozygote: W = 0.92768, *p-*value = 0.5623	Equal variance *F* test *p-*value = 0.178	Unpaired Student’s *t* test	1.766226 to 5.743795	0.002
Fig. 2	*J*	Middle: wild type vs heterozygote	Normal distribution Shapiro–Wilk normality test wild type: W = 0.97022, *p-*value = 0.8939 heterozygote: W = 0.98581, *p-*value = 0.9765	Equal variance *F* test *p-*value = 0.3674	Unpaired Student’s *t* test	1.605875 to 5.699706	0.003
Fig. 2	*J*	Caudal: wild type vs heterozygote	Normal distribution Shapiro–Wilk normality test wild type: W = 0.97971, *p-*value = 0.9501 heterozygote: W = 0.85301, *p-*value = 0.1664	Equal variance *F* test *p-*value = 0.658	Unpaired Student’s *t* test	2.105032 to 6.101853	0.001
Fig. 2	*K*	Rostral: wild type vs heterozygote	Normal distribution Shapiro–Wilk normality test wild type: W = 0.96263, *p-*value = 0.8398 heterozygote: W = 0.95556, *p-*value = 0.7849	Equal variance *F* test *p-*value = 0.3327	Unpaired Student’s *t* test	−203.52664 to 24.79879	0.112
Fig. 2	*K*	Middle: wild type vs heterozygote	Normal distribution Shapiro–Wilk normality test wild type: W = 0.90479, *p-*value = 0.403 heterozygote: W = 0.91236, *p-*value = 0.4521	Equal variance *F* test *p-*value = 0.9113	Unpaired Student’s *t* test	−120.71312 to 31.80191	0.223
Fig. 2	*K*	Caudal: wild type vs heterozygote	Normal distribution Shapiro–Wilk normality test wild type: W = 0.8622, *p-*value = 0.1969 heterozygote: W = 0.89865, *p-*value = 0.366	Equal variance *F* test *p-*value = 0.2966	Unpaired Student’s *t* test	−114.245014 to 4.374468	0.066
Fig. 3	*C*	TBR1 (rostral): wild type vs heterozygote	Normal distribution Shapiro–Wilk normality test wild type: W = 0.87838, *p-*value = 0.3197 heterozygote: W = 0.99953, *p-*value = 0.9585	Unequal variance *F* test *p-*value = 0.03097	Unpaired Welch’s *t* test	−0.7341526 to 0.7142779	0.959
Fig. 3	*C*	TBR1 (middle): wild type vs heterozygote	Normal distribution Shapiro–Wilk normality test wild type: W = 0.95681, *p-*value = 0.6002 heterozygote: W = 0.89516, *p-*value = 0.3703	Equal variance *F* test *p-*value = 0.8262	Unpaired Student’s *t* test	−0.7341526 to 0.7142779	0.543
Fig. 3	*C*	TBR1 (caudal): wild type vs heterozygote	Normal distribution Shapiro–Wilk normality test wild type: W = 0.92614, *p-*value = 0.4743 heterozygote: W = 0.85654, *p-*value = 0.2581	Equal variance *F* test *p-*value = 0.7396	Unpaired Student’s *t* test	−0.7341526 to 0.7142779	0.061
Fig. 3	*C*	RORb (rostral): wild type vs heterozygote	Normal distribution Shapiro–Wilk normality test wild type: W = 0.83679, *p-*value = 0.2057 heterozygote: W = 0.91815, *p-*value = 0.4458	Equal variance *F* test *p-*value = 0.1755	Unpaired Student’s *t* test	−0.7304762 to 0.8176759	0.845
Fig. 3	*C*	RORb (middle): wild type vs heterozygote	Normal distribution Shapiro–Wilk normality test wild type: W = 0.81895, *p-*value = 0.1606 heterozygote: W = 0.92359, *p-*value = 0.4651	Equal variance *F* test *p-*value = 0.666	Unpaired Student’s *t* test	−0.7304762 to 0.8176759	0.870
Fig. 3	*C*	RORB (caudal): wild type vs heterozygote	Normal distribution Shapiro–Wilk normality test wild type: W = 0.85562, *p-*value = 0.2556 heterozygote: W = 0.98158, *p-*value = 0.74	Equal variance *F* test *p-*value = 0.07155	Unpaired Student’s *t* test	−2.202504 to 1.445244	0.486
Fig. 3	*C*	BRN2 (rostral): wild type vs heterozygote	Normal distribution Shapiro–Wilk normality test wild type: W = 0.99286, *p-*value = 0.8385 heterozygote: W = 0.98261, *p-*value = 0.7474	Equal variance *F* test *p-*value = 0.7735	Unpaired Student’s *t* test	−1.8201310 to 0.7466688	0.305
Fig. 3	*C*	BRN2: (middle) wild type vs heterozygote	Non-normal distribution Shapiro–Wilk normality test wild type: W = 0.99043, *p-*value = 0.8129 heterozygote: W = 0.76126, *p-*value = 0.02503	Equal variance *F* test *p-*value = 0.4921	Mann–Whitney *U* test	Z = 0.65465	0.700
Fig. 3	*C*	BRN2 (caudal): wild type vs heterozygote	Normal distribution Shapiro–Wilk normality test wild type: W = 0.984, *p-*value = 0.7578 heterozygote: W = 0.93876, *p-*value = 0.5224	Equal variance *F* test *p-*value = 0.3267	Unpaired Student’s *t* test	−2.011466 to 1.115294	0.413
Fig. 3	*D*	TBR1 (rostral): wild type vs heterozygote	Normal distribution Shapiro–Wilk normality test wild type: W = 0.99311, *p-*value = 0.8413 heterozygote: W = 0.8151, *p-*value = 0.1511	Equal variance *F* test *p-*value = 0.5543	Unpaired Welch’s *t* test	−42.65128 to 80.04344	0.445
Fig. 3	*D*	TBR1 (middle): wild type vs heterozygote	Normal distribution Shapiro–Wilk normality test wild type: W = 0.81232, *p-*value = 0.1443 heterozygote: W = 0.99621, *p-*value = 0.8824	Equal variance *F* test *p-*value = 0.9107	Unpaired Student’s *t* test	−2.011466 to 1.115294	0.215
Fig. 3	*D*	TBR1 (caudal): wild type vs heterozygote	Normal distribution Shapiro–Wilk normality test wild type: W = 0.99479, *p-*value = 0.862 heterozygote: W = 0.90284, *p-*value = 0.3946	Equal variance *F* test *p-*value = 0.362	Unpaired Student’s *t* test	−77.58617 to 17.77541	0.157
Fig. 3	*D*	RORb (rostral): wild type vs heterozygote	Normal distribution Shapiro–Wilk normality test wild type: W = 0.92163, *p-*value = 0.4581 heterozygote: W = 0.94029, *p-*value = 0.5286	Equal variance *F* test *p-*value = 0.2323	Unpaired Student’s *t* test	−101.65007 to 82.43556	0.786
Fig. 3	*D*	RORb (middle): wild type vs heterozygote	Normal distribution Shapiro–Wilk normality test wild type: W = 0.8246, *p-*value = 0.1747 heterozygote: W = 0.95008, *p-*value = 0.5697	Equal variance *F* test *p-*value = 0.8781	Unpaired Student’s *t* test	−95.84147 to 113.25092	0.829
Fig. 3	*D*	RORb (caudal): wild type vs heterozygote	Normal distribution Shapiro–Wilk normality test wild type: W = 0.96848, *p-*value = 0.6591 heterozygote: W = 0.96668, *p-*value = 0.6494	Equal variance *F* test *p-*value = 0.9331	Unpaired Student’s *t* test	−146.25097 to 33.68333	0.157
Fig. 3	*D*	BRN2 (rostral): wild type vs heterozygote	Normal distribution Shapiro–Wilk normality test wild type: W = 0.85, *p-*value = 0.2404 heterozygote: W = 0.88077, *p-*value = 0.3267	Equal variance *F* test *p-*value = 0.9132	Unpaired Student’s *t* test	−259.03986 to 83.37571	0.228
Fig. 3	*D*	BRN2: (middle) wild type vs heterozygote	Normal distribution Shapiro–Wilk normality test wild type: W = 0.90474, *p-*value = 0.4008 heterozygote: W = 0.9984, *p-*value = 0.9235	Equal variance *F* test *p-*value = 0.6548	Unpaired Student’s *t* test	−211.6771 to 138.7905	0.595
Fig. 3	*D*	BRN2 (caudal): wild type vs heterozygote	Normal distribution Shapiro–Wilk normality test wild type: W = 0.98321, *p-*value = 0.7518 heterozygote: W = 0.95459, *p-*value = 0.5899	Equal variance *F* test *p-*value = 0.7567	Unpaired Student’s *t* test	−259.03986 to 83.37571	0.228
Fig. 4	*B*	Rostral: wild type vs heterozygote	Normal distribution Shapiro–Wilk normality test wild type: W = 0.88946, *p-*value = 0.3527 heterozygote: W = 0.90024, *p-*value = 0.3863	Equal variance *F* test *p-*value = 0.3471	Unpaired Student’s *t* test	−0.5520192 to 0.2715238	0.398
Fig. 4	*B*	Middle: wild type vs heterozygote	Normal distribution Shapiro–Wilk normality test wild type: W = 0.97423, *p-*value = 0.6921 heterozygote: W = 0.77489, *p-*value = 0.05587	Equal variance *F* test *p-*value = 0.8878	Unpaired Student’s *t* test	−0.9814349 to 0.2605900	0.182
Fig. 4	*B*	Caudal: wild type vs heterozygote	Normal distribution Shapiro–Wilk normality test wild type: W = 0.96518, *p-*value = 0.6415 heterozygote: W = 0.94166, *p-*value = 0.5341	Equal variance *F* test *p-*value = 0.1779	Unpaired Student’s *t* test	−1.381734 to 0.107321	0.076
Fig. 4	*C*	Rostral: wild type vs heterozygote	Normal distribution Shapiro–Wilk normality test wild type: W = 0.98083, *p-*value = 0.7347 heterozygote: W = 0.99765, *p-*value = 0.9075	Equal variance *F* test *p-*value = 0.9134	Unpaired Student’s *t* test	−334.0189 to 298.0080	0.882
Fig. 4	*C*	Middle: wild type vs heterozygote	Normal distribution Shapiro–Wilk normality test wild type: W = 0.83705, *p-*value = 0.2064 heterozygote: W = 0.95242, *p-*value = 0.5801	Equal variance *F* test *p-*value = 0.6733	Unpaired Student’s *t* test	−157.30316 to 97.03834	0.547
Fig. 4	*C*	Caudal: wild type vs heterozygote	Normal distribution Shapiro–Wilk normality test wild type: W = 0.97936, *p-*value = 0.7246 heterozygote: W = 0.99965, *p-*value = 0.9645	Equal variance *F* test *p-*value = 0.9995	Unpaired Student’s *t* test	−61.38874 to 29.16489	0.379
Fig. 5	*B*	E12.5 BrdU -> P9 fix wild type vs heterozygote	Normal distribution Shapiro–Wilk normality test wild type: W = 0.84955, *p-*value = 0.2247 heterozygote: W = 0.90757, *p-*value = 0.4696	Equal variance *F* test *p-*value = 0.06273	Unpaired Student’s *t* test	−12.9173912 to 0.5582761	0.066
Fig. 5	*B*	E14.5 BrdU -> P9 fix wild type vs heterozygote	Non-normal distribution Shapiro–Wilk normality test wild type: W = 0.78785, *p-*value = 0.08216 heterozygote: W = 0.89427, *p-*value = 0.4031	Unequal variance *F* test *p-*value = 0.03346	Unpaired Welch’s *t* test	−46.46305 to −18.39430	0.004
Fig. 5	*B*	E16.5 BrdU -> P9 fix wild type vs heterozygote	Normal distribution Shapiro–Wilk normality test wild type: W = 0.81206, *p-*value = 0.1257 heterozygote: W = 0.96273, *p-*value = 0.7961	Equal variance *F* test *p-*value = 0.6301	Unpaired Student’s *t* test	−45.14384 to −13.72628	0.004
Fig. 5	*D*	E16.5 BrdU -> P3 fix wild type vs heterozygote	Normal distribution Shapiro–Wilk normality test wild type: W = 0.87183, *p-*value = 0.3007 heterozygote: W = 0.79291, *p-*value = 0.09768	Equal variance *F* test *p-*value = 0.7788	Unpaired Student’s *t* test	−25.481745 to −5.383604	0.013
Fig. 6	*F*	Wild type vs heterozygote (normal position)	Normal distribution Shapiro–Wilk normality test wild type: W = 0.99526, *p-*value = 0.8685 heterozygote (normal position): W = 0.90932, *p-*value = 0.4158	Equal variance Bartlett test *p-*value = 0.0678	Tukey–Kramer test	−437.8421 to 150.364845	0.398
Fig. 6	*F*	Wild type vs heterozygote (ectopic position)	Normal distribution Shapiro–Wilk normality test wild type: W = 0.99526, *p-*value = 0.8685 heterozygote (ectopic position): W = 0.82469, *p-*value = 0.09685	Equal variance Bartlett test *p-*value = 0.0678	Tukey–Kramer test	−514.7175 to −5.315335	0.046
Fig. 6	*F*	Heterozygote (normal position) vs heterozygote (ectopic position)	Normal distribution Shapiro–Wilk normality test heterozygote (normal position): W = 0.90932, *p-*value = 0.4158 heterozygote (ectopic position): W = 0.82469, *p-*value = 0.09685	Equal variance Bartlett test *p-*value = 0.0678	Tukey–Kramer test	−138.4233 to 370.978869	0.443
Fig. 6	*G*	Wild type vs heterozygote (normal position)	Normal distribution Shapiro–Wilk normality test wild type: W = 0.8105, *p-*value = 0.1398 heterozygote (normal position): W = 0.9134, *p-*value = 0.4295	Equal variance Bartlett test *p-*value = 0.3671	Tukey–Kramer test	−6.306416 to 2.5699079	0.496
Fig. 6	*G*	Wild type vs heterozygote (ectopic position)	Normal distribution Shapiro–Wilk normality test wild type: W = 0.8105, *p-*value = 0.1398 heterozygote (ectopic position): W = 0.91542, *p-*value = 0.473	Equal variance Bartlett test *p-*value = 0.3671	Tukey–Kramer test	−6.826894 to 0.8602276	0.131
Fig. 6	*G*	Heterozygote (normal position) vs heterozygote (ectopic position)	Normal distribution Shapiro–Wilk normality test heterozygote (normal position): W = 0.9134, *p-*value = 0.4295 heterozygote (ectopic position): W = 0.91542, *p-*value = 0.473	Equal variance Bartlett test *p-*value = 0.3671	Tukey–Kramer test	−2.728482 to 4.9586403	0.707
Fig. 6	*H*	Wild type vs heterozygote (normal position)	Normal distribution Shapiro–Wilk normality test wild type: W = 0.98032, *p-*value = 0.7312 heterozygote (normal position): W = 0.97948, *p-*value = 0.7255	Unequal variance Bartlett test *p-*value = 0.0005376	Steel–Dwass test		1.000
Fig. 6	*H*	Wild type vs heterozygote (ectopic position)	Normal distribution Shapiro–Wilk normality test wild type: W = 0.98032, *p-*value = 0.7312 heterozygote (ectopic position): W = 0.92588, *p-*value = 0.5487	Unequal variance Bartlett test *p-*value = 0.0005376	Steel–Dwass test		0.256
Fig. 6	*H*	Heterozygote (normal position) vs heterozygote (ectopic position)	Normal distribution Shapiro–Wilk normality test heterozygote (normal position): W = 0.97948, *p-*value = 0.7255 heterozygote (ectopic position): W = 0.92588, *p-*value = 0.5487	Unequal variance Bartlett test *p-*value = 0.0005376	Steel–Dwass test		0.256
Fig. 7	*E*	Wild type vs heterozygote	Normal distribution Shapiro–Wilk normality test wild type: W = 0.98136, *p-*value = 0.7384 heterozygote: W = 0.82619, *p-*value = 0.1787	Equal variance *F* test *p-*value = 0.1953	Unpaired Student’s *t* test	−27.896256 to 6.444708	0.158
Fig. 7	*F*	Wild type vs heterozygote	Normal distribution Shapiro–Wilk normality test wild type: W = 0.98334, *p-*value = 0.7528 heterozygote: W = 0.79961, *p-*value = 0.1136	Equal variance *F* test *p-*value = 0.5522	Unpaired Student’s *t* test	−18.92532 to 75.37736	0.172
Fig. 7	*G*	L1: wild type vs heterozygote	Normal distribution Shapiro–Wilk normality test wild type: W = 0.89302, *p-*value = 0.3636 heterozygote: W = 0.89602, *p-*value = 0.3729	Equal variance *F* test *p-*value = 0.9608	Unpaired Student’s *t* test	49.88475 to 142.24042	0.004
Fig. 7	*G*	L2: wild type vs heterozygote	Normal distribution Shapiro–Wilk normality test wild type: W = 0.95785, *p-*value = 0.6051 heterozygote: W = 0.8297, *p-*value = 0.1876	Equal variance *F* test *p-*value = 0.1826	Unpaired Student’s *t* test	−113.18916 to −22.48398	0.021
Fig. 7	*H*	Wild type vs heterozygote	Non-normal distribution Shapiro–Wilk normality test wild type: W = 0.76125, *p-*value = 0.02501 heterozygote: W = 0.77171, *p-*value = 0.0486		Mann–Whitney *U* test	Z = −0.65465	0.700
Fig. 7	*I*	L1: wild type vs heterozygote	Normal distribution Shapiro–Wilk normality test wild type: W = 0.80732, *p-*value = 0.1321 heterozygote: W = 0.998, *p-*value = 0.9147	Equal variance *F* test *p-*value = 0.6222	Unpaired Student’s *t* test	2.056330 to 4.188658	0.001
Fig. 7	*I*	L2: wild type vs heterozygote	Non-normal distribution Shapiro–Wilk normality test wild type: W = 0.80371, *p-*value = 0.1234 heterozygote: W = 0.96722, *p-*value = 0.6523	Equal variance *F* test *p-*value = 0.2364	Unpaired Student’s *t* test	−6.311426 to −0.491803	0.032
Extended Data Fig. 3-1	*B*	Rostral: wild type vs heterozygote	Non-normal distribution Shapiro–Wilk normality test wild type: W = 0.85766, *p-*value = 0.1813 heterozygote: W = 0.82895, *p-*value = 0.1053	Equal variance *F* test *p-*value = 0.7773	Unpaired Student’s *t* test	−0.0421572482 to 0.0007444148	0.057
Extended Data Fig. 3-1	*B*	Middle: wild type vs heterozygote	Non-normal distribution Shapiro–Wilk normality test wild type: W = 0.88419, *p-*value = 0.2889 heterozygote: W = 0.92997, *p-*value = 0.5799	Equal variance *F* test *p-*value = 0.1479	Unpaired Student’s *t* test	−0.033846035 to −0.002103765	0.030
Extended Data Fig. 3-1	*B*	Caudal: wild type vs heterozygote	Non-normal distribution Shapiro–Wilk normality test wild type: W = 0.93585, *p-*value = 0.6259 heterozygote: W = 0.90844, *p-*value = 0.4262	Equal variance *F* test *p-*value = 0.9502	Unpaired Student’s *t* test	−0.030801905 to −0.009642128	0.002
Extended Data Fig. 3-1	*C*	Rostral: wild type vs heterozygote	Non-normal distribution Shapiro–Wilk normality test wild type: W = 0.90901, *p-*value = 0.4299 heterozygote: W = 0.93146, *p-*value = 0.5914	Unequal variance *F* test *p-*value = 0.02958	Unpaired Welch’s *t* test	−164.72649 to 42.39315	0.199
Extended Data Fig. 3-1	*C*	Middle: wild type vs heterozygote	Non-normal distribution Shapiro–Wilk normality test wild type: W = 0.93329, *p-*value = 0.6057 heterozygote: W = 0.97804, *p-*value = 0.9414	Equal variance *F* test *p-*value = 0.1447	Unpaired Student’s *t* test	−187.04365 to −24.62301	0.016
Extended Data Fig. 3-1	*C*	Caudal: wild type vs heterozygote	Non-normal distribution Shapiro–Wilk normality test wild type: W = 0.82064, *p-*value = 0.08938 heterozygote: W = 0.80381, *p-*value = 0.06359	Equal variance *F* test *p-*value = 0.6413	Unpaired Student’s *t* test	−232.61149 to 44.94482	0.163
Extended Data Fig. 3-1	*D*	Rostral: wild type vs heterozygote	Non-normal distribution Shapiro–Wilk normality test wild type: W = 0.71196, *p-*value = 0.008242 heterozygote: W = 0.93999, *p-*value = 0.6591		Mann–Whitney *U* test	Z = −0.64051	0.589
Extended Data Fig. 3-1	*D*	Middle: wild type vs heterozygote	Non-normal distribution Shapiro–Wilk normality test wild type: W = 0.87287, *p-*value = 0.2379 heterozygote: W = 0.94396, *p-*value = 0.6912	Equal variance *F* test *p-*value = 0.8054	Unpaired Student’s *t* test	−953.6398 to 772.9249	0.820
Extended Data Fig. 3-1	*D*	Caudal: wild type vs heterozygote	Non-normal distribution Shapiro–Wilk normality test wild type: W = 0.89833, *p-*value = 0.3642 heterozygote: W = 0.68099, *p-*value = 0.003873		Mann–Whitney *U* test	Z = −0.2445	0.853
Extended Data Fig. 3-2		TBR1: wild type vs heterozygote	Non-normal distribution Shapiro–Wilk normality test wild type: W = 0.93052, *p-*value = 0.4906 heterozygote: W = 0.8333, *p-*value = 0.1967	Equal variance *F* test *p-*value = 0.3502	Unpaired Student’s *t* test	−50.99473 to 10.79854	0.145
Extended Data Fig. 3-2		RORB: wild type vs heterozygote	Non-normal distribution Shapiro–Wilk normality test wild type: W = 0.77427, *p-*value = 0.05444 heterozygote: W = 0.90745, *p-*value = 0.4096	Equal variance *F* test *p-*value = 0.1612	Unpaired Student’s *t* test	−38.81443 to −12.39362	0.006
Extended Data Fig. 3-2		Brn2: wild type vs heterozygote	Non-normal distribution Shapiro–Wilk normality test wild type: W = 0.9922, *p-*value = 0.8311 heterozygote: W = 0.87014, *p-*value = 0.2959	Equal variance *F* test *p-*value = 0.7895	Unpaired Student’s *t* test	−57.77120 to 86.12194	0.609
Extended Data Fig. 4-1	*A*	Wild type vs heterozygote	Non-normal distribution Shapiro–Wilk normality test wild type: W = 0.97442, *p-*value = 0.9297 heterozygote: W = 0.77181, *p-*value = 0.009691		Mann–Whitney *U* test	Z = −1.7228	0.089
Extended Data Fig. 4-1	*B*	Wild type vs heterozygote	Non-normal distribution Shapiro–Wilk normality test wild type: W = 0.96529, *p-*value = 0.8517 heterozygote: W = 0.85836, *p-*value = 0.09203	Equal variance *F* test *p-*value = 0.7867	Unpaired Student’s *t* test	−22.37795 to 178.75553	0.119
Extended Data Fig. 4-1	*C*	Wild type vs heterozygote	Non-normal distribution Shapiro–Wilk normality test wild type: W = 0.93988, *p-*value = 0.5806 heterozygote: W = 0.96297, *p-*value = 0.8289	Equal variance *F* test *p-*value = 0.3549	Unpaired Student’s *t* test	−20.29773 to 17.85329	0.894
Extended Data Fig. 4-1	*D*	Wild type vs heterozygote	Non-normal distribution Shapiro–Wilk normality test wild type: W = 0.90943, *p-*value = 0.3119 heterozygote: W = 0.96112, *p-*value = 0.8099	Equal variance *F* test *p-*value = 0.6479	Unpaired Student’s *t* test	−75.85538 to 144.40307	0.519
Extended Data Fig. 4-1	*E*	Wild type vs heterozygote	Non-normal distribution Shapiro–Wilk normality test wild type: W = 0.88971, *p-*value = 0.1982 heterozygote: W = 0.97785, *p-*value = 0.9523	Equal variance *F* test *p-*value = 0.944	Unpaired Student’s *t* test	−47.06784 to 102.62339	0.443
Extended Data Fig. 4-1	*F*	Wild type vs heterozygote	Non-normal distribution Shapiro–Wilk normality test wild type: W = 0.8859, *p-*value = 0.181 heterozygote: W = 0.86775, *p-*value = 0.1163	Equal variance *F* test *p-*value = 0.6467	Unpaired Student’s *t* test	−244.6362 to 902.2204	0.242

**Figure 1.**
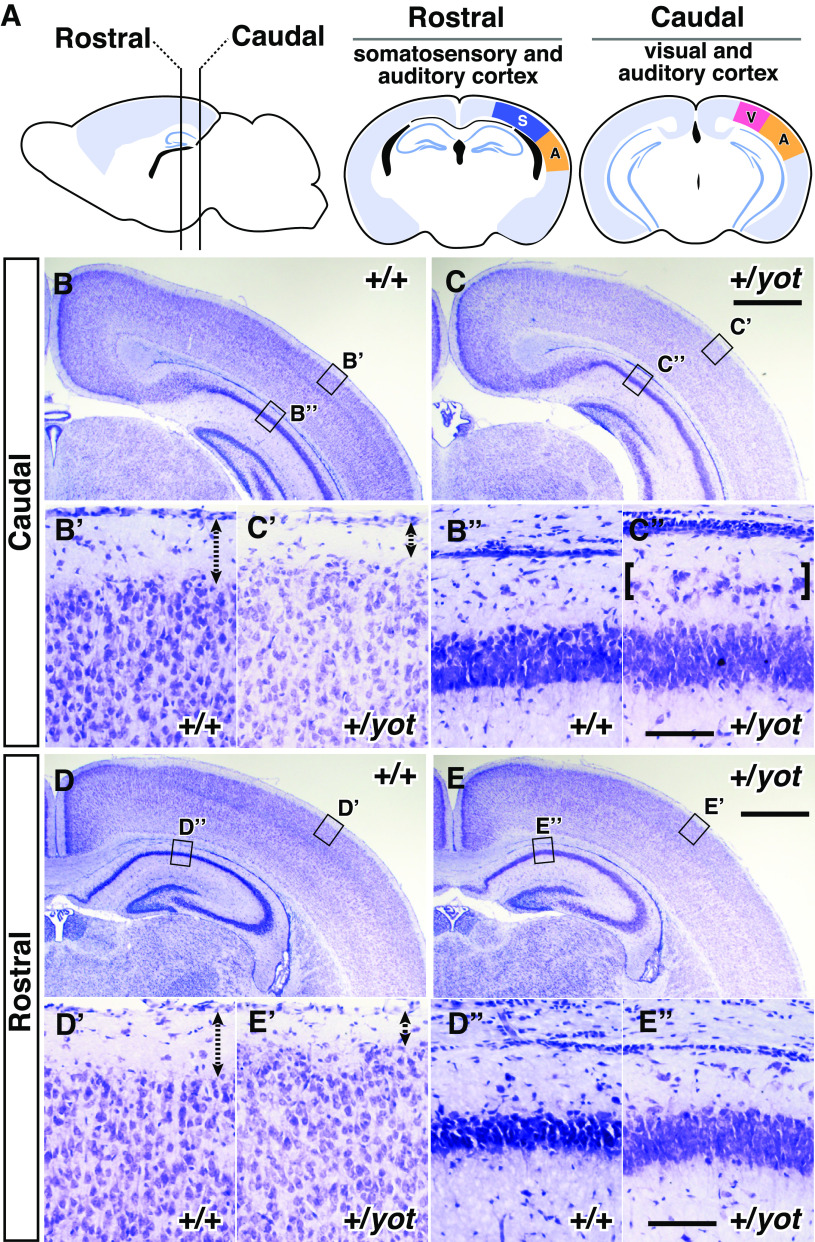
Cortical layer 1 is thinner and the CA1 pyramidal cell layer is split in the caudal hippocampus of heterozygous *yotari* mutant mice. ***A***, Schematic illustrations of the analyzed brain regions in ***B–E’’***. S: somatosensory cortex; A: auditory cortex; V: visual cortex. ***B–E’’***, Nissl-stained coronal brain sections of the caudal region containing the visual and auditory cortices (***B–C’’***, Caudal) and rostral region containing the somatosensory and auditory cortices (***D–E’’***, Rostral) from wild-type mice (+/+; ***B***, ***B’***, ***B’’***, ***D***, ***D’***, ***D’’***) and heterozygous *yotari* mice (+/*yot*; ***C***, ***C’***, ***C’’***, ***E***, ***E’***, ***E’’***) at P7. Enlarged views from the regions enclosed by rectangles in ***B***, ***C***, ***D***, ***E*** are shown in ***B’***, ***B’’***, ***C’***, ***C’’***, ***D’***, ***D’’***, ***E’***, ***E’’***, respectively. ***B’*** and ***C’*** show auditory cortex, and ***D’*** and ***E’*** show somatosensory cortex. Layer 1 is shown by dashed arrows with two ends (***B’***, ***C’***, ***D’***, ***E’***). Ectopic cells in heterozygous *yotari* mutant mice are indicated by brackets in ***C’’***. Scale bars: 1 mm (***B***, ***C***, ***D***, ***E***) and 10 μm (***B’***, ***B’’***, ***C’***, ***C’’***, ***D’***, ***D’’***, ***E’***, ***E’’***).

10.1523/ENEURO.0433-22.2023.f1-1 Extended Data Figure 1-1 Nissl staining of the cerebral cortex at P0. Nissl-stained coronal sections of cerebral cortex from wild-type (+/+) and heterozygous *yotari* (+/*yot*) mice obtained at P0. Scale bar: 1 mm. Download Figure 1-1, EPS file.

**Figure 2.**
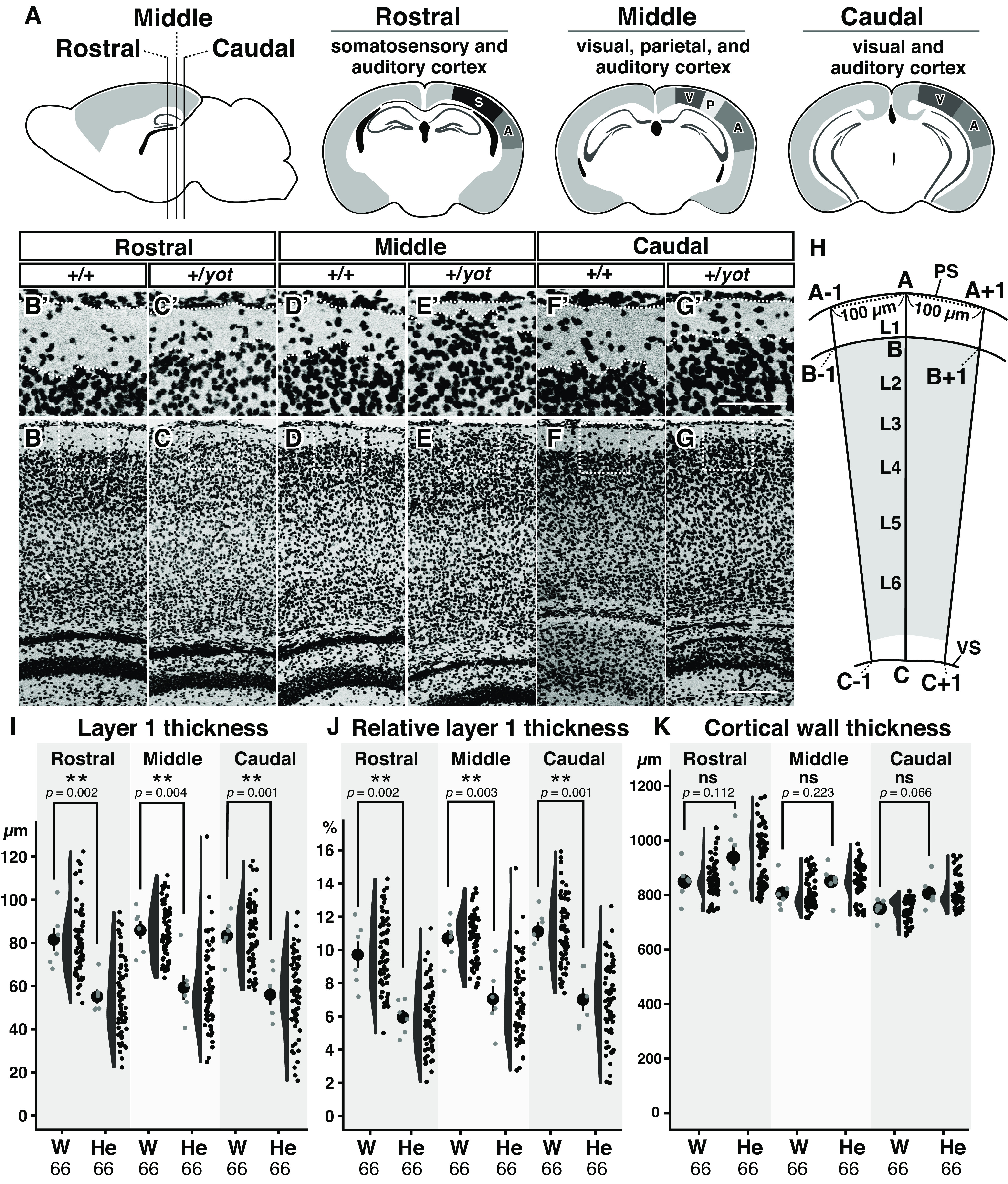
Reduced neocortical layer 1 thickness in heterozygous *yotari* mutant mice. ***A***, Schematic illustrations showing brain regions analyzed for layer 1 and cortical wall thickness. S: somatosensory cortex; A: auditory cortex; V: visual cortex, P: parietal cortex. ***B–G’***, DAPI staining of wild-type (+/+; ***B***, ***B’***, ***D***, ***D’***, ***F***, ***F’***) and heterozygous *yotari* (+/*yot*, ***C***, ***C’***, ***E***, ***E’***, ***G***, ***G’***) mouse cerebral neocortex at P7. Representative images obtained from the somatosensory (Rostral: ***B***, ***B’***, ***C***, ***C’***), parietal (Middle: ***D***, ***D’***, ***E***, ***E’***), and visual (Caudal: ***F***, ***F’***, ***G***, ***G’***) cortex. Boxed regions in ***B–G*** are magnified in ***B’–G’***, respectively. ***H***, Schematic illustration showing a region of the cerebral neocortex. Straight lines from points A, A − 1, and A + 1 on the pial surface (PS) to points C, C − 1, and C + 1 on the ventricular surface (VS), and their intersections B, B − 1, and B + 1, respectively, between layer 1 (L1) and layer 2 (L2). See Materials and Methods and Extended Data Figure 2-1 for details. ***I–K***, Combination of dot, violin, and scatter plots showing layer 1 thickness (***I***), relative layer 1 thickness (***J***), and cortical wall thickness (***K***) across brain regions. The black dot and bar in the leftmost dot plot indicate mean and SD, respectively, and each gray dot indicates the mean from one individual brain. Each black circle in the scatter plot is a raw datapoint. Measurement was performed on six brains obtained from six different litters (*n *=* *6). Three different coronal sections were obtained from the caudal, middle, and rostral brain of each mouse as shown in ***A***, and 11 positions were analyzed per brain region. Mean values were then calculated for each brain region. Genotype differences were analyzed by Student’s *t* test. **p <* *0.01, ns (not significant) *p *>* *0.05. Scale bars: 200 μm (***B–G***) and 50 μm (***B’–G’***).

10.1523/ENEURO.0433-22.2023.f2-1 Extended Data Figure 2-1 Schematic illustrations showing the three brain regions along the rostro-caudal axis and the 11 positions analyzed in each brain region. Schematic illustrations of the three brain regions analyzed for layer 1 and cortical wall thickness, the rostral region containing the somatosensory and auditory cortices (Rostral), middle brain region containing the visual, parietal, and auditory cortices (Middle), and the caudal brain region containing the visual and auditory cortices (Caudal). In the lower panel, the 11 positions analyzed in each brain region (A, A + 1–5, A − 1–5) are shown by dashed lines. S: somatosensory cortex; A: auditory cortex; V: visual cortex, P: parietal cortex. Download Figure 2-1, EPS file.

### Measurements of relative cell position and distance from the ventricular surface

**Figure 3.**
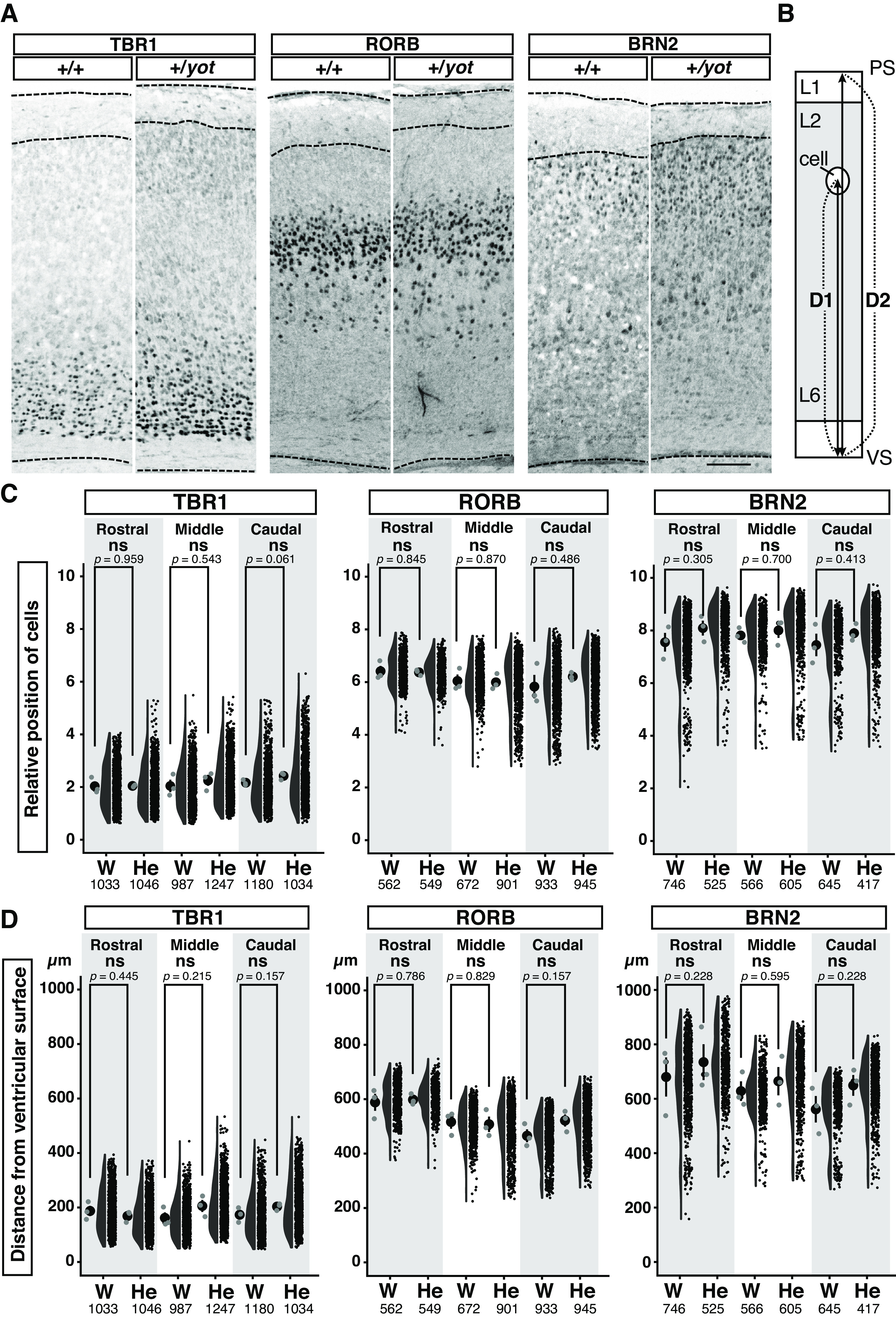
Half reduction of the *Dab1* gene dosage does not cause a significant positional change of neocortical layer 2–6 neurons. ***A***, TBR1-positive, RORB-positive, or BRN2-positive cells in the somatosensory (Rostral), parietal (Middle), and visual (Caudal) cortices of P7 wild-type (+/+) and heterozygous *yotari* mice (+/*yot*). Representative images of coronal neocortical sections in the middle brain region are shown. ***B***, Schematic illustration showing a region of the cerebral neocortex. Distances from the ventricular surface (VS) to the target cells (D1) and cortical wall thickness (D2) were measured, and the relative positions of cells were calculated by dividing D1 by D2 and multiplying by 10. ***C***, ***D***, Combination of dot, violin, and scatter plots showing the relative positions of layer marker-positive cells (***C***) and distances of layer marker-positive cells from the ventricular surface (***D***) within three brain regions along the rostro-caudal axis. The black dot and bar in the leftmost dot plot indicate the mean and SD, respectively, and each gray dot indicates the mean of one brain. Each black circle in the scatter plot is a raw datapoint. Measurements were performed on three brains from different litters (*n *=* *3) per group. The total number of analyzed cells (pooled from three mice per group) are shown below each graph. After calculation of mean values for each brain, most group differences were analyzed by independent samples Student’s *t* test; however, some experiments were analyzed by Mann–Whitney *U* test [***C***, BRN2 (Middle)] or unpaired Welch’s *t* test [***C***, TBR1 (Rostral); ***D***, TBR1 (Rostral)]. The *p*-values are labeled on the graph. ns (not significant) *p *>* *0.05. Scale bar: 100 μm.

**Figure 4.**
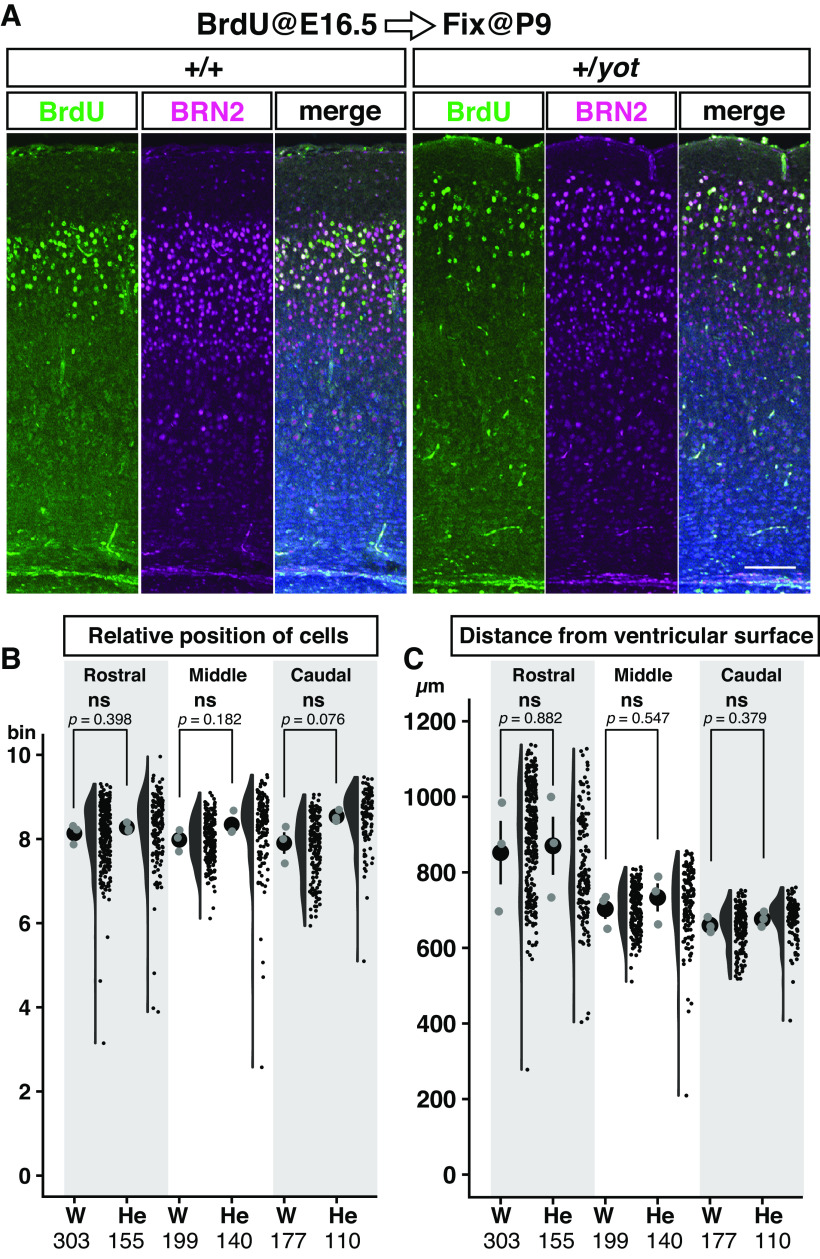
There were no significant positional changes in late-born, BRN2-positive, superficial neocortical neurons in *yotari* heterozygous mice. ***A***, Coronal sections of P9 parietal neocortex from wild-type (+/+) and heterozygous *yotari* (+/*yot*) mice injected with BrdU at E16.5. Sections were co-stained with BrdU (green), the layer 2 marker BRN2 (magenta), and the nuclear stain DAPI (blue). ***B***, ***C***, Combination of dot, violin, and scatter plots showing relative positions of BrdU/BRN2 double-positive cells between the ventricular surface and pial surface (***B***) and distances of BrdU/BRN2 double-positive cells from the ventricular surface (***C***) in somatosensory (Rostral), parietal (Middle), and visual (Caudal) cortices of wild-type (W) and heterozygous *yotari* (He) mice. The black dot and bar in the leftmost dot plot indicate the mean and SD, respectively, each gray dot indicates the mean from one brain, and each black circle in the scatter plot is a raw datapoint. Data were obtained from three brains per group, each from different litters (*n *=* *3). Total analyzed cell numbers pooled from three mice per group are shown below each graph. After calculation of the mean values for each brain, group differences were analyzed by independent samples Student’s *t* test. The *p*-values are labeled on the graph. ns (not significant). Scale bar: 100 μm.

Distances from the ventricular surface to marker-positive cells (D1; see Fig. 3*B*), distances from the ventricular surface to the pial surface through each of these cells (D2; see Fig. 3*B*), and distances from the ventricular surface to BrdU/BRN2 double-positive cells (Fig. 4) were measured using Fiji software. Relative position was then calculated as D1/D2 × 10. These measurements were performed on three brains obtained from three different litters, and results were compared between groups by independent samples Student’s *t* test, unpaired Welch’s *t* test, or Mann–Whitney *U* test using the R language environment as described in Table 1.

10.1523/ENEURO.0433-22.2023.f3-1 Extended Data Figure 3-1 The neocortical region from layer 2 to the ventricular surface of heterozygous *yotari* mice is expanded and the number of cells is increased but there is no change in the cell density. ***A***, Schematic showing the dorsolateral cerebral neocortex. The measurement area was from the boundary of layer 1 (L1) and layer 2 (L2) to the ventricular surface (VS; 200-μm width, dark gray area). PS: pial surface; L6: layer 6. ***B–D***, Sections of the rostral brain region containing the somatosensory and auditory cortex (Rostral), middle brain region containing the visual, parietal, and auditory cortex (Middle), and caudal brain region containing the visual and auditory cortex (Caudal) from six wild-type (W) and heterozygous *yotari* mice (He) each, all from different litters. Confocal images were then taken of the somatosensory, parietal, and visual cortex, respectively. Numbers of DAPI-positive cells within the measurement region are shown in ***C*** and cell density in ***D*** on combination dot and scatter plots. The black dots with a black bar indicate the mean and SD, respectively, and each gray dot is a raw datapoint. The total numbers of analyzed mice are shown below each graph. Statistical significance was evaluated by independent samples Student’s *t* test (***B–D***: Middle) or Mann–Whitney *U* test (***D***: Rostral and Caudal). The *p*-values are labeled on the graph. *p <* *0.05, ** *p *<* *0.01, ns (not significant) *p *>* *0.05. Download Figure 3-1, EPS file.

10.1523/ENEURO.0433-22.2023.f3-2 Extended Data Figure 3-2 Heterozygous *yotari* mice exhibit an expanded RORB-positive cell layer compared to wild-type mice. ***A***, Schematic illustration showing how layer thickness was defined as the distance from the 20th to 80th percentile of layer marker-positive cells. ***B***, Thicknesses of TBR1-positive, RORB-positive, or BRN2-positive cell layers measured on coronal sections of the somatosensory (Rostral), parietal (Middle), and visual (Caudal) cortex from three wild-type (W) and three heterozygous *yotari* mice (He), each of different litters. The black dot and bar in the leftmost dot plot indicate the mean and SD, respectively, each gray dot indicates the mean from one brain, and each black dot in the scatter plot is a datapoint obtained from the rostral, middle, or caudal brain regions. The total number of analyzed coronal brain sections is shown at the bottom of the graph. Groups were compared by independent samples Student’s *t* test. The *p*-values are labeled on the graph. **p <* *0.01, ns (not significant) *p *>* *0.05. Download Figure 3-2, EPS file.

### Measurement of neocortical layer thickness by the distribution of layer marker-positive cells

For quantitative comparisons, we defined layer thickness as the distance between the 20th and 80th percentile of layer marker-positive cells (Extended Data Fig. 3-2*A*). The markers used were TBR1, RORB, or BRN2. Measurement was performed using coronal sections of somatosensory, parietal, and visual cortices obtained from three brains from different litters. Then the average thickness of each brain along the rostro-caudal axis was calculated. Results were compared between groups by independent samples Student’s *t* test using the R language environment.

### Measurement of pyramidal neuron apical dendrite angle in cerebral neocortex and hippocampus

**Figure 6.**
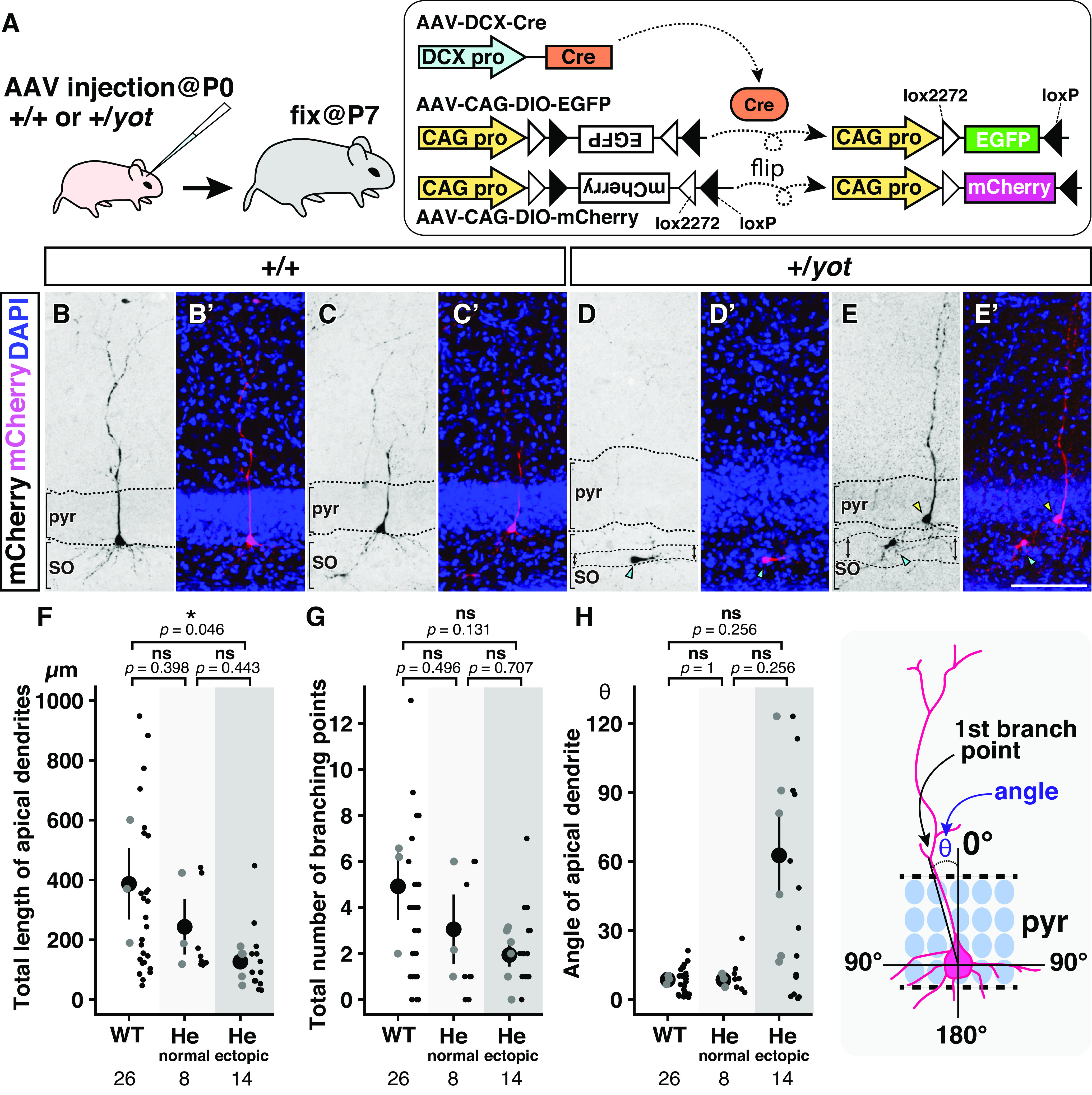
Ectopic cells within the split pyramidal cell layer have shorter and misoriented apical dendrites. ***A***, Schematic illustration showing the time schedule and strategy for sparse labeling of hippocampal cells. A mixture of AAV-DCX-Cre, AAV-CAG-DIO-EGFP, and AAV-CAG-DIO-mCherry was injected into the lateral ventricles of wild-type and heterozygous *yotari* mice at P0, and brain slices were prepared at P7. AAV-DCX-Cre expresses Cre under the control of a DCX promoter (DCX pro), while AAV-CAG-DIO-EGFP and AAV-CAG-DIO-mCherry contain a DIO-EGFP or DIO-mCherry cassette driven by a CAG promoter (CAG pro). The DIO cassette is composed of two reciprocally oriented loxP sites (black triangles) and two lox2272 sites (white triangles), and EGFP, or mCherry cDNA was inserted within the DIO cassette in the opposite direction to the CAG promoter; therefore, there is no transcription of those cDNA under normal conditions. However, simultaneous infection with Cre recombinase flips the orientation of EGFP or mCherry cDNA into the forward direction by two-step recombination between loxP and lox2272 sites. The enclosed rectangle and rounded rectangle represent the Cre cDNA and Cre protein, respectively. ***B–E’***, Coronal sections of caudal neocortex (including visual and auditory cortex) from wild-type (+/+, ***B–C’***) and heterozygous *yotari* (+/*yot*, ***D–E’***) mice were stained with anti-RFP (black or magenta) and DAPI (blue). Representative images of mCherry-positive cells in the pyramidal cell layer (pyr) of wild-type mice (***B–C’***), normally distributed cells in the pyramidal cell layer of heterozygous *yotari* mice (yellow arrowheads in ***E*** and ***E’***), and ectopically distributed cells in the SO (cyan arrowheads in ***D–E’***) of heterozygous *yotari* mice (***D–E’***). The ectopic cell regions in the SO of heterozygous *yotari* mice are indicated by double-headed arrows (***D*** and ***E***). ***F–H***, Total length of apical dendrites (***F***), total number of branching points (***G***), and angle of apical dendrites (***H***) obtained for 26 neurons from three wild-type (WT) mice, eight normally distributed neurons from three heterozygous *yotari* mice (He, normal), and 14 ectopically distributed neurons from six heterozygous *yotari* mice (He, ectopic) are plotted by a combination of dot and scatter plots. All analyzed mice were obtained from different litters. The large black dot and the bar in the leftmost dot plot indicate the mean and SD, respectively, and the gray dots indicate the mean from one brain. Each small black dot in the scatter plot is a raw datapoint. The total number of analyzed cells is shown below each graph. After calculation of the mean values from each brain, groups were compared by Tukey–Kramer test (***F*** and ***G***) or Steel–Dwass test (***H***). The *p*-values are labeled on the graph. *p <* *0.05, ns (not significant) *p *>* *0.05. The schematic drawing in H shows a pyramidal neuron in the pyramidal cell layer (pyr). The angle (θ) of the primary apical dendrite was determined as the angle between a perpendicular line passing through the center of the neuron and the first branch point.

**Figure 7.**
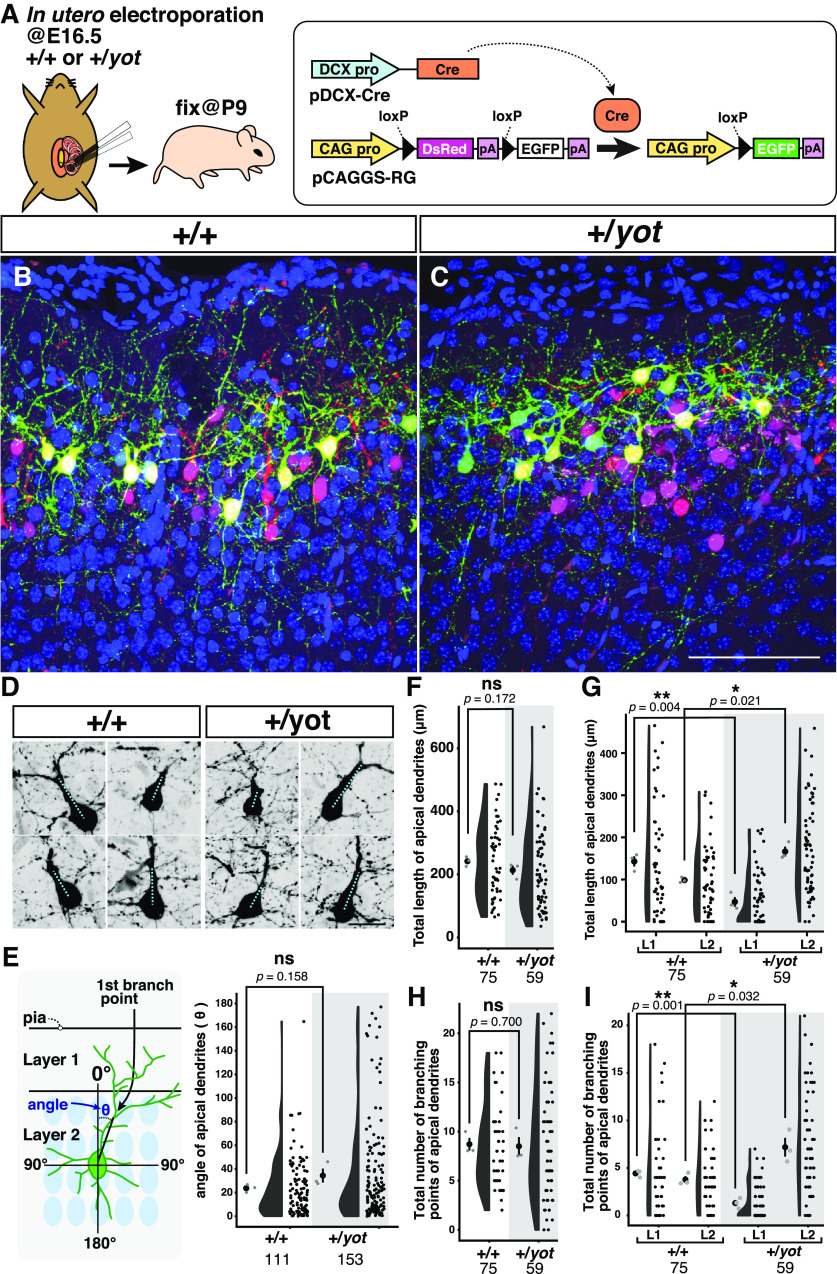
Superficial late-born neocortical neurons of heterozygous *yotari* mice show less apical dendrite sprouting in layer 1. ***A***, Strategy and time schedule for sparse labeling of superficial neurons. The DCX promoter-driven Cre expression plasmid pDCX-Cre was co-electroporated with the Cre-reporter plasmid pCAGGS-RG at E16.5, and mice were fixed at P9. In the absence of Cre, the pCAGGS-RG expresses DsRed under the CAG promoter, whereas in the presence of Cre, the floxed-DsRed-poly A expression cassette is excised and the plasmid expresses EGFP. To reduce the recombination probability, pDCX-Cre plasmid concentration was significantly reduced. DCX pro, CAG pro, and pA represent the DCX promoter, CAG promoter, and simian virus 40 polyadenylation signal, respectively. The rectangle and rounded rectangle represent the Cre cDNA and Cre protein, respectively. ***B***, ***C***, Electroporated brains at P9 obtained from wild-type (***B***, +/+) and heterozygous *yotari* mice (***C***, *+/yot*) were sectioned coronally. The superficial part of the somatosensory cortex is shown. Scale bar: 100 μm. ***D***, Sparsely labeled EGFP-positive cells obtained from wild-type (+/+) and heterozygous *yotari* mice (+/*yot*) for measurement of primary apical dendrite angle. Cyan dotted lines are drawn from the center of the cell body to the first branch point. Scale bar: 20 μm. ***E***, A schematic illustrating a neuron in the superficial cerebral neocortex. The angle of the primary apical dendrite (θ) was defined as the angle between a perpendicular line passing through the center of the cell and the first branch point. ***E–G***, Combination of dot, violin, and scatter plots showing the apical dendrite angle (***E***), total length (***F***), and total lengths specifically in layer 1 (L1) and layer 2 (L2) (***G***). ***H***, ***I***, The total number of apical dendrite branching points (***H***) and the total number of branching points within layer 1 (L1) and layer 2 (L2) (***I***) obtained from three WT (+/+) and six heterozygous *yotari* mice (*+/yot*) from different litters. The black dot and bar in the leftmost dot plot indicate the mean and the SD, respectively, and the gray dots indicates the mean for each brain. Each black circle in the scatter plot is a raw datapoint. The total number of analyzed cells is shown below each graph. After calculation of mean values for each brain, groups were compared by independent samples Student’s *t* test (***E***, ***F***, ***G***, ***I***) or Mann–Whitney *U* test (***H***). The *p*-values are labeled on the graph. *p <* *0.05, **p <* *0.01, ns (not significant) *p *>* *0.05.

To measure the apical dendrite angle of pyramidal neurons in the cerebral neocortex, a perpendicular line was drawn from the pial surface to the center of the target cell body, from which another straight line was drawn to the first branch point of the apical dendrite (Fig. 7*D*,*E*). The angle between these two straight lines was measured using Fiji software and defined as the apical dendrite angle. To measure the apical dendrite angle of hippocampal pyramidal neurons, a line perpendicular to the pyramidal cell layer was drawn to the center of the target cell body and another from the target cell body center to the first branch point of the apical dendrite (Fig. 6*H*). The angle between the lines was then measured using Fiji software. Measurements were conducted on more than three brains, all obtained from different litters. Angles were compared between groups by the Steel–Dwass test using the R language environment as described in Table 1.

### Measurement of apical dendrite length and number of branching points within neocortical layer 1 or layers 2

Sparsely labeled EGFP-positive cells using electroporation as described above were selected, and their apical dendrite length and number of apical dendrite branching points within layer 1 and layer 2 were measured using Fiji software, respectively. Brains were obtained from more than three mice, each from different litters, and results compared between groups by independent samples Student’s *t* test or Mann–Whitney *U* test using the R language environment as described in Table 1.

### Measurement of the total apical dendrite length and total number of branching points in the hippocampus

Total apical dendrite length and branch point number were determined using Fiji software. Measurements were performed using more than three brains derived from different litters, and results compared between groups by Tukey–Kramer test using the R language environment as described in Table 1.

### Experimental design and statistical analysis

Experimental design details, sample sizes, and results of statistical analyses are presented in the corresponding Materials and Methods and Results sections, figure legends, and Table 1. Mice of either sex were used throughout the study. Exact *p*-values are listed on Table 1 and also labeled on figures. A *p* < 0.05 was considered significant for all tests.

## Results

### Heterozygous *yotari* mutant mice exhibit a thinner neocortical layer 1 than wild-type mice and a split CA1 pyramidal cell layer in the caudal hippocampus

To examine whether neuronal positioning is disrupted by reduced *Dab1* gene expression, we compared the cytoarchitecture of the cerebral neocortex and hippocampus between wild-type and heterozygous *yotari* mutant mice using Nissl staining at P0 and P7. There were no obvious differences in cortical layer structure between genotypes at P0 (Extended Data Fig. 1-1), but layer 1 was significantly thinner in both the rostral brain region containing somatosensory and auditory cortices (Fig. 1, Rostral) and caudal brain region containing visual and auditory cortices (Fig. 1, Caudal) of P7 heterozygous *yotari* mutant mice compared with P7 wild-type mice.

To quantitatively evaluate potential differences in neocortical layer thickness between wild-types and heterozygous *yotari* mutant mice, we stained nuclei in coronal brain sections with DAPI (Fig. 2*B–G*,*B’–G’*) and measured layer 1 thickness (Fig. 2*I*) and cortical wall thickness (Fig. 2*K*; see Materials and Methods). Consistent with Nissl staining, layer 1 thickness (Fig. 2*I*) of the rostral brain region containing somatosensory and auditory cortices (Fig. 2; Extended Data Fig. 2-1, Rostral), middle brain region containing visual, parietal, and auditory cortices (Fig. 2; Extended Data Fig. 2-1, Middle), and caudal brain region containing visual and auditory cortices (Fig. 2; Extended Data Fig. 2-1, Caudal) were significantly reduced in *yotari* heterozygous mice compared with wild-type mice. Moreover, relative layer 1 thickness, calculated as the ratio of layer 1 thickness to cortical wall thickness, was also reduced in rostral, middle, and caudal regions of heterozygous *yotari* mouse neocortex (Fig. 2*J*). In the hippocampus as well, layer structure was abnormal in heterozygous *yotari* mice, but was manifested by an abnormal split in the caudal CA1 pyramidal cell layer (Fig. 1*C*,*C’’*).

### The RORB-positive layer 4 but not TBR1-positive and BRN2-positive layers 5/6 and 2 are expanded in the neocortex of heterozygous *yotari* mice

The reduced layer 1 thickness observed among heterozygous *yotari* mice by Nissl and DAPI staining may result from disruption of the cortical layer structure and/or positional changes in cortical neurons (as seen in *reeler* and *yotari* homozygous mutants; Howell et al., 1997; Sheldon et al., 1997; Yoneshima et al., 1997; Trommsdorff et al., 1999). To distinguish between these possibilities, we examined whether relative position or distance from the ventricular surface of cortical neurons is differed between genotypes by staining coronal brain sections with antibodies specifically targeting layer 5/6 neurons (TBR1), layer 4 neurons (RORB), or layer 2/3 neurons (BRN2), and measured individual cell positions (Fig. 3). While there were no significant differences in relative position of cells (Fig. 3*C*), scatter plots revealed that BRN2-positive layer 2/3 neurons tended to be located farther from the ventricular surface in heterozygous *yotari* mice than wild-type mice (Fig. 3*D*), suggesting that layer 2/3 is shifted by cell dispersion into layer 1.

We then examined whether this reduction in layer 1 thickness was accompanied by changes in other layers. Briefly, we defined a 200-μm wide measurement area from the layer 1–2 boundary to the ventricular surface (Extended Data Fig. 3-1*A*) and examined layer 2/3, 4, and 5/6 area (Extended Data Fig. 3-1*B*), cell number (Extended Data Fig. 3-1*C*), and cell density (Extended Data Fig. 3-1*D*) in somatosensory (Rostral), parietal (Middle), and visual (Caudal) cortices. We found that the measured area was significantly larger in the middle and caudal brain regions of heterozygous *yotari* mice (Extended Data Fig. 3-1*B*). There was also a significant increase in cell number within the middle brain region of heterozygous *yotari* mice, and a numerical increase in both rostral and caudal brain regions that did not reach statistical significance (Extended Data Fig. 3-1*C*). In contrast, there were no differences in cell density between wild-type and heterozygous *yotari* mutant mice (Extended Data Fig. 3-1*D*).

We also examined the thickness of each layer by measuring the distributions of neurons immunopositive for layer-specific markers (Extended Data Fig. 3-2). For comparison, we defined the layer thickness as the length along which the 20th–80th percentile population of layer marker-positive cells is distributed (Extended Data Fig. 3-2*A*) as it was difficult to clearly determine the boundaries of layers based on the distribution of the all layer marker positive cells. This analysis revealed no significant difference in TBR1-positive layer 5/6 thickness or BRN2-positive layer 2/3 thickness, but RORB-positive layer 4 thickness was greater in heterozygous *yotari* mice than wild-type mice (Extended Data Fig. 3-2*B*). These results suggest that a half reduction in the *Dab1* gene dosage causes a decrease in layer 1 thickness as well as an increase in the area of the neocortical region from layer 2 to the ventricular surface and cell number with an expansion of RORB-positive layer 4 thickness.

To further explore whether the reduction of DAB1 protein causes superficial positional shift of the superficial layer neurons, late-born neurons were labeled by BrdU at E16.5 and visualized at P9 (Fig. 4*A*) together with BRN2 immunostaining in somatosensory (Rostral), parietal (Middle), and visual (Caudal) cortices (Fig. 4*B*,*C*). There was no significant genotype difference in either mean relative position of BrdU/BRN2 double-positive neurons (Fig. 4*B*) or mean distance from the ventricular surface (Fig. 4*C*). Similarly, there were no significant differences in the number of E16.5-BrdU-labeled cells, BRN2-positive cells, and BrdU/BRN2 double-positive cells in a 200-μm-wide region from the layers 1–2 boundary to the ventricular surface between wild-type and heterozygous *yotari* mice (Extended Data Fig. 4-1).

10.1523/ENEURO.0433-22.2023.f4-1 Extended Data Figure 4-1 There were no significant changes in number and cell density of E16.5-BrdU-labeled cells, BRN2-positive cells, and BrdU/BRN2 double-positive cells between wild-type and heterozygous *yotari* mice. The numbers of BrdU/BRN2 double-positive cells (***A***), E16.5-BrdU-labeled cells (***C***), and BRN2-positive cells (***E***) were counted in a 200-μm-wide region between the layer 1–2 boundary and ventricular surface (shown in Extended Data Fig. 3-1*A*). Corresponding cell densities were then calculated (***B***, ***D***, ***F***). Measurements were performed in the somatosensory (rostral), parietal (middle), and the visual (caudal) cortex of three wild-type (W) and three *yotari* heterozygous mice (He), each from different litters. The large black dots and black bars in the graph represent the mean and SD, respectively. The gray dots indicate the mean from one brain and each small black dot in the scatter plot is a raw datapoint obtained from the rostral, middle, or caudal brain region. The total number of analyzed brain sections is shown at the bottom of the graph. Groups were compared by Mann–Whitney *U* test (***A***) or independent samples Student’s *t* test (***B–F***). The *p*-values are labeled on the graph. ns (not significant) *p *>* *0.05. Download Figure 4-1, EPS file.

Collectively, these results confirmed that a reduction in *Dab1* gene expression does not severely disrupt the basic layer structure or layer-specific spatial distribution of neocortical neurons. In addition, these findings suggest that expression from a single *Dab1* gene is sufficient for the radial migration of late-born neurons, and that the reduction in layer 1 thickness is not caused by disruption of radial migration of the neocortical neurons.

### Abnormal splitting of the CA1 pyramidal layer in heterozygous *yotari* mice is caused mainly by migration failure of late-born neurons

**Figure 5.**
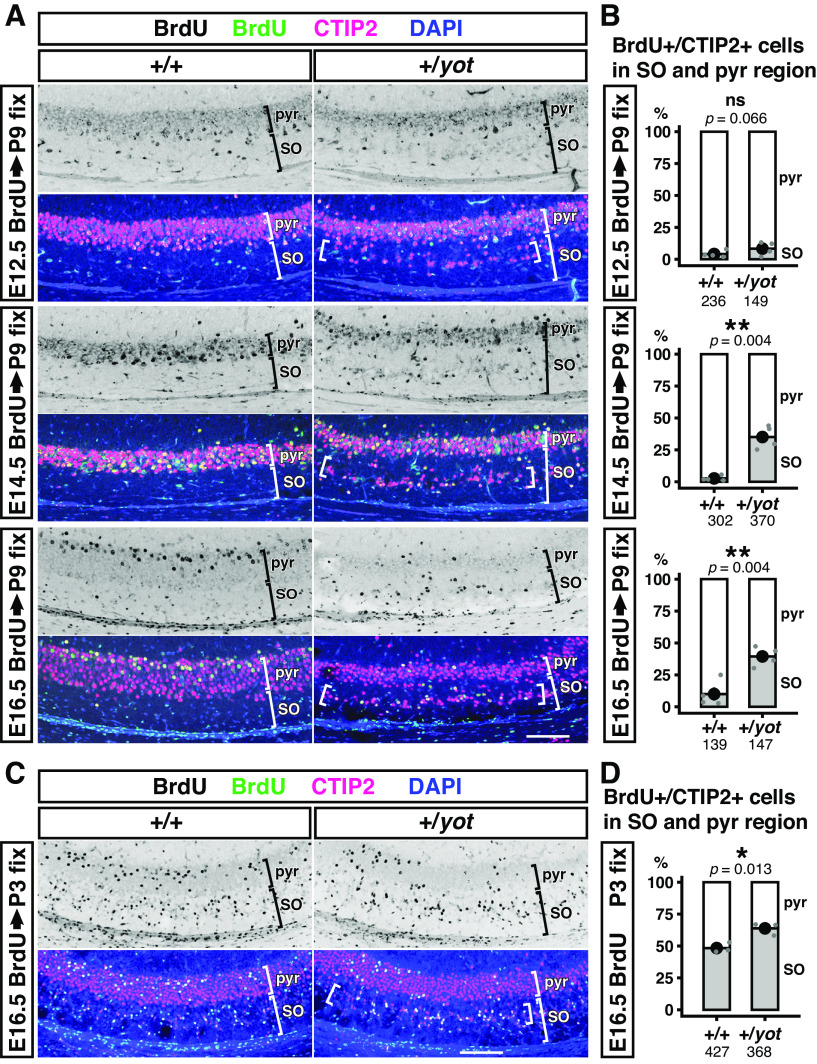
Abnormal splitting of the CA1 pyramidal cell layer in the caudal hippocampus of heterozygous *yotari* mice is due mainly to migration failure of later-born neurons. ***A***, ***C***, BrdU was administered at E12.5, E14.5, or E16.5 to wild-type (+/+) and heterozygous *yotari* (+/*yot*) mice, and brains were fixed at P9 (***A***) or P3 (***C***). Coronal sections of the caudal brain region containing the visual and auditory cortex were stained using anti-BrdU antibody (black or green) and anti-CTIP2 antibody (magenta), while nuclei were stained with DAPI (blue). Abnormal splitting of the CA1 pyramidal cell layer is indicated by the region between two brackets in stratum oriens. SO, stratum oriens; pyr, pyramidal cell layer. ***B***, ***D***, Proportions (%) of BrdU/CTIP2 double-positive cells within the SO and pyramidal cell layer (pyr) shown by 100% stacked bar chart and dot plots. The black dots within the bar indicate the mean percentage of four (***B***) or three (***D***) brains from different litters (*n *=* *4 or 3), and the SD is shown by the black bars. Each gray dot indicates the mean from one brain. The number of analyzed cells is shown below each graph. After calculation of mean values from each brain, group differences were analyzed by independent samples Student’s *t* test (***B***, BrdU labeling at E12.5 in the wild-type vs heterozygote, BrdU labeling at E16.5 in the wild-type vs heterozygote, and ***D***) or Welch’s unpaired *t* test (***B***, BrdU labeling at E14.5 in the wild-type vs heterozygote). The *p*-values are labeled on the graph. *p <* *0.05, **p <* *0.01, ns (not significant) *p *>* *0.05. Scale bar: 100 μm.

Nissl staining revealed an abnormally split CA1 cell layer in the caudal hippocampus of P7 heterozygous *yotari* mice (Fig. 1*C*,*C’’*). To investigate the identity of the abnormally located cells and to determine their birthdates, mice were injected with BrdU at E12.5, E14.5, and E16.5, and brains were fixed for co-staining with the CA1 neurons marker CTIP2 at P9 (Fig. 5*A*). Subsequent analysis revealed that almost all abnormally located cells were CTIP2-positive and most were produced in the late stages of development as shown by greater BrdU staining at E16.5 compared with earlier times (Fig. 5*A*,*B*).

We then examined whether splitting of the CTIP2-positive pyramidal cell layer is caused by failure of neuronal migration. According to the GFP-labeling study, E16.5-born neurons labeled with GFP by *in utero* electroporation migrate within the stratum oriens (SO) around P0–P3 and reach the top of the pyramidal cell layer at P4 (Kitazawa et al., 2014). Thus, we examined the positioning of E16.5 BrdU-labeled and CTIP2-positive cells at P3. We found that more E16.5 BrdU-labeled CTIP2-positive cells were distributed in the SO of heterozygous *yotari* mice than wild-type mice at P3 (Fig. 5*D*). These results suggest that abnormal splitting of the pyramidal cell layer occurs during migration within the SO and is already started at P3 (region shown with brackets in Fig. 5*C* +/*yot*).

### Mispositioned hippocampal pyramidal neurons in heterozygous *yotari* mice have shorter and misoriented apical dendrites

Previous studies have shown that null mutation of the *Dab1* gene impairs pyramidal neuron development and reduces dendritic complexity in the hippocampus (Niu et al., 2004; Matsuki et al., 2008). Here, we examined whether reduced *Dab1* also results in abnormal apical dendrite morphology by sparse *in utero* labeling of hippocampal pyramidal neurons using a DIO reporter system (Sohal et al., 2009; Fig. 6*A*). Briefly, a reporter gene (GFP or mCherry) is inserted within the DIO cassette in reverse orientation with respect to the promoter, thereby preventing translation under normal conditions but allowing translation in the presence of Cre recombinase as the reporter gene orientation is switched. Further, to induce DIO-mediated reporter gene expression specifically in neurons, Cre expression was controlled by a promoter for *Doublecortin* (*Dcx*), a gene thought to be expressed predominantly in newly generated neurons, including hippocampal pyramidal neurons (X. Wang et al., 2007; Yoo et al., 2011). Thus, we co-injected rAAV vectors carrying (1) a Cre recombinase driven by a *Dcx* promoter (AAV-DCX-Cre), (2) DIO-EGFP driven by a CAG promoter (AAV-CAG-DIO-EGFP), or (3) DIO-mCherry driven by a CAG promoter (AAV-CAG-DIO-mCherry) to wild-type and heterozygous *yotari* mice at P0, and prepared brain sections at P7. For sparse labeling, concentrations of AAV-CAG-DIO-EGFP and AAV-CAG-DIO-mCherry were appropriately reduced based on prior examinations. We then compared apical dendrite morphology among (1) mCherry-positive cells distributed within the pyramidal cell layer of wild-type mice (Fig. 6*B–C’*), (2) mCherry-positive cells normally distributed within the pyramidal cell layer of heterozygous *yotari* mice (Fig. 6*E*,*E’*, yellow arrowheads), and (3) mCherry-positive cells ectopically distributed within the SO of heterozygous *yotari* mice (Fig. 6*D–E’*, cyan arrowheads). Total apical dendrite length was significantly shorter among ectopically positioned neurons in heterozygous *yotari* mice compared with wild-types (Fig. 6*F*). Total apical dendritic length was also numerically reduced among normally positioned neurons in heterozygous *yotari* mice compared with wild types, although the difference did not reach statistical significance. In addition, the total number of branching points was numerically reduced among normally positioned and ectopically positioned neurons of heterozygous *yotari* mice compared with wild-types (Fig. 6*G*). Furthermore, many of the primary apical dendrites in ectopically positioned neurons of heterozygous *yotari* mice were orientated in abnormal directions, although the average did not differ significantly between genotypes (Fig. 6*H*).

### Neocortical layer 2 pyramidal neurons in heterozygous *yotari* mice tend to extend their apical dendrites within layer 2 rather than in layer 1

In addition to a migration defect, null mutation, or knock-down of *Dab1* causes hypomorphic growth and misorientation of apical dendrites in the cerebral neocortex (Niu et al., 2004; Olson et al., 2006; Franco et al., 2011). Layer 1 is composed mainly of the apical dendrites projecting from pyramidal neurons in layers 2/3 and 5, suggesting that the thinner layer 1 documented here may stem from fewer apical dendrites reaching into the superficial cortex. To simultaneously observe positioning of neurons and the fine dendritic morphology, we introduced a plasmid DNA solution containing pCAGGS-RG, which expresses DsRed under the control of a CAG promoter, and pDCX-Cre into late-born superficial neurons by *in utero* electroporation at E16.5 (Fig. 7*A*). Since the DsRed cassette is flanked by two loxP sites, in the presence of Cre, the DsRed cassette is removed and EGFP begins to be transcribed. By reducing the concentration of pDCX-Cre, we sparsely labeled neurons with EGFP. For quantitative analysis of apical dendrite morphology, we first measured the angle between the first branch point (the end of the main shaft of the apical dendrite) and the perpendicular line passing through the center of the cell body (Fig. 7*D*,*E*) but found no significant difference between genotypes (Fig. 7*E*). Similarly, there were no genotype differences in total length and total number of branching points (Fig. 7*F*,*H*). However, we found significant differences both in the total length of apical dendrites and the total number of branching points when measured separately within layers 1 and 2 (Fig. 7*G*,*I*). Those apical dendrites within layer 1 were significantly shorter and less morphologically complex in heterozygous *yotari* mutant mice than wild-type mice, while those within layer 2 were longer and more branched in heterozygous *yotari* mutant mice. These results suggest that a reduction of DAB1 protein disrupts the normal guidance of apical dendrites.

## Discussion

We demonstrate that *Dab1* is haploinsufficient for the formation of the cerebral neocortex and hippocampus, although specific developmental processes are differentially sensitive to *Dab1* gene dose. Neocortical layer 1 is thinner in heterozygous *yotari* mutant mice than in wild-type mice (Figs. 1 and 2), but layer marker staining and BrdU birth-dating indicated that half reduction of *Dab1* does not disrupt radial neuronal migration and layer formation (Figs. 3 and 4). Rather, sparse labeling revealed that layer 2 neurons tend to elongate apical dendrites within layer 2 rather than layer 1 (Fig. 7). Heterozygous *yotari* mutant mice also exhibit splitting of the CA1 pyramidal cell layer in the caudal hippocampus (Figs. 1 and 5), and BrdU birth-dating studies suggests that this developmental abnormality is caused, at least in part, by migration defects of late-born pyramidal neurons (Fig. 5). Further, sparse labeling revealed that mispositioned neurons in the SO tend to have short and abnormally orientated dendrites (Fig. 6). Thus, reduction of *Dab1* gene expression has distinct effects on development of the cerebral neocortex and hippocampus.

Homozygous null mutation of the *Dab1* gene causes severe migration failure both in the cerebral neocortex and hippocampus (Howell et al., 1997; Yoneshima et al., 1997; Franco et al., 2011; Blume et al., 2017). However, half reduction of *Dab1* gene expression did not cause migration failure in the cerebral neocortex, and impaired only late-born pyramidal neuron migration in the caudal hippocampus. This regional difference may be attributable to distinct migration modes. Early-born pyramidal neurons in the cerebral neocortex migrate radially by somal translocation, while late-born neurons follow a series of steps including multipolar migration, locomotion, and terminal translocation (Noctor et al., 2001; Tabata and Nakajima, 2003; Tabata et al., 2009; Sekine et al., 2011; Ohtaka-Maruyama and Okado, 2015; Kon et al., 2017). In contrast, hippocampal pyramidal neurons migrate radially by a climbing mode after a multipolar stage (Kitazawa et al., 2014; Hayashi et al., 2015). These various migration modes may have different DAB1 protein sensitivities (requirements), although it is still unclear why only the caudal hippocampus is affected as migration modes are similar across the hippocampal rostral–caudal axis. It may be that a subpopulation of pyramidal neurons in caudal hippocampus, possibly comprising a specific sublayer, requires both *Dab1* gene copies for proper migration in the caudal hippocampus (Soltesz and Losonczy, 2018).

Homozygous null mutation of the *Dab1* gene also causes complete loss of cerebral neocortex layer 1 (Sweet et al., 1996; Howell et al., 1997; Ware et al., 1997; Yoneshima et al., 1997). In contrast to this severe phenotype, half reduction of *Dab1* gene resulted only in a thinner layer 1 throughout the rostral–caudal axis (Figs. 1 and 2). This abnormality appears to arise after radial neuronal migration is complete, as no obvious anatomic change in neocortical structure was observed at P0 (Extended Data Fig. 1-1), migration failure was not observed (Fig. 4), and layer-specific marker staining showed no positional changes in TBR1-positive, RORB-positive, or BRN2-positive cells (Fig. 3). These effects of heterozygous *Dab1 yotari* mutation further suggest two distinct signaling pathways for excitatory neurons with differential dependencies on *Dab1* gene dosage: a less sensitive pathway for radial neuronal migration and a sensitive pathway for the control of layer 1 thickness.

Several other mutant mouse lines exhibit abnormal and ectopic positioning of cells in cortical layer 1, including *Dab1^p45/–^* (Herrick and Cooper, 2002), *Reelin ΔC-KI* (Kohno et al., 2015), *Vldlr* KO (Hack et al., 2007; Hirota and Nakajima, 2020), *Apoer2* KO (Hirota et al., 2018), *DeltaNp73^Cre^;ROSA26^stop-dt-a^* (de Frutos et al., 2016), *Emx1^Cre^;Wnt3a^dt-a^* (de Frutos et al., 2016), *Emx1-Cre;Pcdh-γ^fcon3/fcon3^* (Garrett et al., 2012), *Plexin A2/A4* double KO (Hatanaka et al., 2019), and *Sema6a* KO (Hatanaka et al., 2019) mice. Many of these lines harbor mutations in the Reelin signaling pathway. The *Dab1^p45^* gene encodes a hypomorphic alternative splice variant of *Dab1,* and *Dab1^p45/–^* mice also show thinning of layer 1 and splitting of the CA1 pyramidal layer (Herrick and Cooper, 2002). Since the *Dab1^p45^* allele was created by the insertion of *Dab1^p45^* cDNA, only the DAB1 p45 protein is expressed in this mouse (Herrick and Cooper, 2002). Therefore, the *Dab1^p45/–^* condition is distinct from the heterozygous null condition, and analysis of heterozygous *Dab1* mutant mice such as *yotari* could be particularly valuable for elucidating the pathomechanisms of human diseases associated with heterozygous *Dab1* loss-of-function mutations. While it is yet unknown whether haploinsufficiency of the *Dab1* gene has similar effects on human cortical and hippocampal development, haploinsufficiency of the related *Reelin* gene is associated with various neuropsychiatric disorders (Guidotti et al., 2016; Ishii et al., 2016; Hattori and Kohno, 2021). Given the critical role of *Dab1* in the canonical Reelin signaling pathway (Honda et al., 2011; Jossin, 2020), the findings of this study could provide potential explanations for human diseases.

Similar to heterozygous *yotari* mice, *Reelin ΔC-KI* mouse lacking the C-terminal region of Reelin, which is thought to be required for efficient DAB1 phosphorylation (Nakano et al., 2007), gradually show thinning of layer 1 and apical dendrites with abnormal orientations that tend to avoid growing into the layer 1 (Kohno et al., 2015). The *DeltaNp73^Cre^;ROSA26^stop-dt-a^* and the *Emx1^Cre^;Wnt3a^dt-a^* mouse lines also underexpress Reelin protein and show reduced layer 1 thickness and cortical neurons with decreased apical dendrite tuft complexity (de Frutos et al., 2016). Since the Reelin receptor VLDLR is predominantly expressed in the superficial cerebral neocortex (Hirota et al., 2015), *Vldlr* KO would result in deficient Reelin signaling within superficial cortical layers, and indeed *Vldlr* KO also results in ectopic positioning of CUX1-positive or CUX2-positive neurons in layer 1 (Hack et al., 2007; Hirota and Nakajima, 2020). Therefore, it appears that partial attenuation of the Reelin-Dab1 signaling pathway does not inhibit radial neuronal migration in the cerebral neocortex but causes a decrease in the layer 1 thickness.

We suggest that these deficits account at least in part for the reduction in layer 1 thickness. Layer 1 is composed of the apical dendrites of excitatory neurons within layers 2/3 and 5, local interneurons, and Cajal-Retzius cells, thalamic axons, and axons of excitatory neurons projecting from other layers and regions of the cerebral neocortex (Solari and Stoner, 2011; Harris and Shepherd, 2015; Genescu and Garel, 2021; Gesuita and Karayannis, 2021; Schuman et al., 2021). However, there is a particularly strong association between apical dendrites and layer 1 thickness as evidenced by (1) the tendency of apical dendrites projecting from sparsely labeled superficial layer neurons in heterozygous *yotari* mice to elongate within layer 2 but not layer 1 (Fig. 7), (2) the reduced layer 1 thickness and associated apical dendrite maldevelopment among Reelin C-terminal deletion mutant mice (Kohno et al., 2015), Reelin-producing Cajal-Retzius cell-ablated mice (de Frutos et al., 2016), and *Dab1* knock-down or knock-out experiments (Olson et al., 2006; Franco et al., 2011; Sekine et al., 2011, 2012), and (3) defects in apical dendrite morphology observed in other mutants with abnormal layer 1 reduction, such as *Emx1-Cre;Pcdh-γ^fcon3/fcon3^* (Garrett et al., 2012), *PlexinA2/A4* double KO, and *Sema6a* KO mice (Hatanaka et al., 2019). Thus, abnormalities in the apical dendrites of neocortical pyramidal neurons may explain reduced layer 1 thickness. It is also possible that the increase in cell number within other neocortical layers (Extended Data Fig. 3-1) leads to a reduction in layer 1 thickness. The causal relationship between the increase in cell number and the expansion of the neocortical area, and why the cell number increased, are important subjects for future study.

In addition to abnormal development of neocortical layer 2 apical dendrites, we also observed a decrease in the total length of apical dendrites projecting from ectopic pyramidal neurons of the caudal hippocampus as well as misoriented apical dendrites among subset of neurons. While it is possible that the half reduction of DAB1 protein directly caused these phenotypes (Niu et al., 2004; Matsuki et al., 2008), it is also possible that these abnormalities are secondary to migration failure or that neurons ectopically distributed in the SO region are still migrating and these misoriented structures are leading processes rather than dendrites. Regardless of mechanism, the resulting maldevelopment in the hippocampus may contribute to a myriad of neuropsychiatric diseases exhibiting deficits in learning and memory formation (Hainmueller and Bartos, 2020).

Similar to the *Dab1* gene, the *Reelin* gene is also known to exhibit haploinsufficiency (Liu et al., 2001; Carboni et al., 2004; Qiu et al., 2006; Nullmeier et al., 2011; Bouamrane et al., 2016; L. Wang et al., 2019), and reduced Reelin levels have been observed in the brains of patients with schizophrenia (Impagnatiello et al., 1998; Guidotti et al., 2000), bipolar disorder (Guidotti et al., 2000), autism (Fatemi et al., 2001, 2005), and Alzheimer’s disease (Chin et al., 2007). Moreover, heterozygous *reeler* mice and *Reelin ΔC-KI* mice exhibit similar neurochemical, behavioral, and cognitive abnormalities to those observed in autism or schizophrenia (Costa et al., 2001; Tremolizzo et al., 2002; Carboni et al., 2004; Nullmeier et al., 2011; Sakai et al., 2016; Sánchez-Hidalgo et al., 2022). The anatomic abnormalities we observed in heterozygous *yotari* mice may also be related to behavioral defects because layer 1 and hippocampus are essential for higher-order cognitive functions (Hainmueller and Bartos, 2020; Schuman et al., 2021). Consistent with this possibility, behavioral abnormalities observed in schizophrenia, such as hyperactivity, decreased anxiety-like behavior, and impaired working memory, have been observed in dorsal forebrain-specific *Dab1* conditional knock-out mice (Imai et al., 2017). These findings suggest that therapeutic enhancement of Reelin signaling could prevent or ameliorate the cognitive deficits of schizophrenia and other neurodevelopmental disorders (Guidotti et al., 2016; Ishii et al., 2016; Hattori and Kohno, 2021). Indeed, administration of Reelin to mouse models of cognitive dysfunction such as Angelman syndrome and schizophrenia could prevent or ameliorate the cognitive deficits. (Hethorn et al., 2015; Ishii et al., 2015; Ibi et al., 2020; Sawahata et al., 2021). If a similar neuropsychiatric abnormality is observed because of *Dab1* haploinsufficiency, it may be possible to ameliorate the defect by activating the *Dab1* signaling through Reelin supplementation.

In conclusion, while the radial migration of neocortical neurons can proceed normally when *Dab1* is reduced to half, *Dab1* is haploinsufficient for maintaining layer 1 thickness and for the migration of late-born pyramidal neurons in the caudal hippocampus. Further studies are required to explore why these developmental events are differentially dependent on *Dab1* expression level. We suggest that elucidation of the Reelin signaling pathways may provide clues to neuropsychiatric disease pathogenesis and potential therapeutic strategies.

## Synthesis

Reviewing Editor: Lynda Erskine, University of Aberdeen Decisions are customarily a result of the Reviewing Editor and the peer reviewers coming together and discussing their recommendations until a consensus is reached. When revisions are invited, a fact-based synthesis statement explaining their decision and outlining what is needed to prepare a revision will be listed below. The following reviewer(s) agreed to reveal their identity: Alessandra Pierani. Note: If this manuscript was transferred from JNeurosci and a decision was made to accept the manuscript without peer review, a brief statement to this effect will instead be what is listed below.

Following review of your manuscript by 2 experts in the field, both are agreed that your manuscript makes an important contribution to the field. They have suggestions for improving the manuscript prior to publication, all of which can be addressed by modifications to the text or figures. Below is a synthesis of the review indicating the points you may wish to consider in revising the manuscript prior to publication. These are aimed at further helping readers to understand the data and its significance to the field. Thank you for submitting your interesting work to eNeuro.

General comments from reviewers:

The authors very carefully performed an extensive set of morphological examinations on cortical and hippocampal pyramidal neurons in the heterozygous yotari mouse, and revealed *Dab1* haploinsuffisiency in their developmental behaviors. Cell visualization and tracking were nicely done, and the interpretations sound reasonable.

This study assesses the role of *Dab1* gene dosage in cerebral cortex development. DAB1 encodes for an adaptor protein known to be a key regulator of Reelin signalling in the control of neuronal migration and positioning in the developing brain. *Dab1* has been the subject of much research but the mechanisms mediating the reelin-Dab1 signalling pathway are yet not fully elucidated, including whether Reelin function depends on Dab1dosage. The authors examined *Dab1* haploinsufficiency by using the heterozygous yotari mouse model, which is an autosomal recessive null mutant of Dab1. Altogether, the authors were able to show convincingly that 1) *Dab1* is haploinsufficient for maintaining the thickness of layer 1 and dendrite guidance of L2 pyramidal neurons in the cerebral cortex, although radial migration is not altered; 2) based on IHC and biirthdating radial migration of late-born pyramidal neurons in the caudal hippocampus is sensitive to *Dab1* dosage and heterozygous yotari display SP splitting, branching and misoriented dendrites.

This is an interesting and important question in the field and the experiments were well planned and executed. The manuscript is well written and the images are of very good quality. Only minor corrections are required, mainly to help the readers understand the data and further discuss the results.

Minor points to consider:

1. A brief discussion of whether the “subtle attenuation of the Reeling-Dab1 signaling pathway”, surely found in heterozygous yotari, could potentially be treated by REELIN exogenously administrated would be fruitful.

2. It would be helpful to mention/discuss, in the introduction and/or discussion, the relevance and impact that this study could bring for the clinics. Which pathologies are described to be caused by *Dab1* mutations, are they heterozygous or homozygous? The discussion of these literatures would strengthen the significance of this study.

3. Introduction

- Line 46: missing Falconer 1951 reference.

- line 52: ref Niu 2004 it’s a study in hippocampus, not in neocortex

- lines 64/65: don’t understand the meaning of the sentence “Various studies of haploinsufficiency suggested that genes which show haploinsufficiency have some tendency for its functions.”

- Line 87 authors say “than in other brain regions”. Which other brain regions are these? Were they analysed in this study?

4. Results

- Figure 1: authors describe comparisons in neocortex and hippocampal structures at P7 at 2 brain levels, one caudal at the level of visual cortex, and another rostral at the level of motor cortex (line 319). However, the images in Fig.1C-D do not represent motor cortex, which should be much more rostral. The close-ups C’ and D’ are in fact S1-S2 cortex. Similarly, the authors mention visual cortex for the caudal level, but the close-ups A’ and B’ represent auditory cortex. Even if the authors wanted to mention visual cortex just as a reference for the selected caudal section, why not mentioning auditory cortex since that is what is shown in the close-ups?

- Figure 2: Same comment as before concerning the brain areas represented in images A-F according to the description in the figure legend. Is A-B motor cortex, based on the images sections chosen in figure 1C-D? The authors say that 3 different sections were obtained: caudal (visual), middle (auditory), rostral (motor) cortex, so sections mean “brain regions”? I would consider sections as slice sections in the rostrocaudal axis, and the visual and auditory cortex would be in the same caudal section. I think this part can be a little confusing for the reader to understand which sections/brain areas were used in each analysis. These descriptions should be rethought.

Moreover, in the Materials and Methods line 242, it’s mentioned that the analysis was done in a region of SS cortex but the figure legend indicates that 11 positions were measured per section (visual, auditory, motor). So, I couldn’t really understand what region(s) the data represent.

The authors show a clear reduction in L1 thickness in yotari mice (fig2H) but the relative thickness is ns, although almost, which is surprising since the cortical wall thickness is not different. Maybe some regions present more differences in thickness than others and by pooling all data these differences might be lost. Showing this analysis separated by brain region could be insightful.

- Figure 3 author say: “we found that Brn2-positive layer 2/3 neurons in yotari heterozygous mice tended to locate more distantly from the VS than those in wt”. Authors assess their position using BrdU birthdating assays showing no changes in the pattern and position of these neurons. However, it’s true that in graph D and in images E, L2/3 looks more extended and cells more disperse, as if L2/3 was pushed up because L1 is thinner. Is this observed at all brain levels analysed? And about cell number/density? Are the number of Brn2+ or BrdU+ alone the same? And number of BrdU+/Brn2+? Could the thickness of individual layers be altered?

Regarding Rorb, the distribution of each cell in graph C in WT is not the same as in graph D in WT, unlike in the mutant and for the other markers. Could the authors explain why that is?

Brain section/level for images in Fig.3A should be mentioned in results and figure legend.

- Figure 4: what about the percentage of cells in the pyr layer, is it changed in the mutant? Was this analysed?

Substitute “BCL11B” in lines 363 and 810. Bcl11b is the name of the gene, Ctip2 is the protein.

- Figure 5: wrong figure reference in line 406.

Are the mispositioned neurons in SO still migrating neurons? The horizontal orientation of the cell soma seems to indicate that.

Authors describe a tendency in yotari mice for having shorter total length of apical dendrites because neurons in both pyr and SO layers were analysed. Neurons in SO look either immature or migrating still, but looking at neurons is pyr layer, is this tendency still observed? They look very similar when comparing images E with images B-C.

- Figure 6: in panel B-C, it looks like the cortex in +/yot is bigger than WT and it was shown no differences in the cortical wall length (Fig.2J), so this representation can be a little misleading to the reader.

Authors say in line 429-430 “in the heterozygous yotari mice the apical dendrites of layer 2 neurons do not appear to be directed in the layer 1 (Fig. 6C)”. It’s too of a strong affirmation, since is still possible to observe apical dendrites in L1. The frequency of sprouting into L1 was not analysed.

5. Discussion

- Line 487: authors hypothesize that reduction of L1 in het yotari might be caused after neurons finish their radial migration and due to the abnormalities in their apical dendrites that probably do not sustain the further development of L1. This is a very interesting point, that is supported by many other mouse models with similar phenotypes, and which the authors discuss extensively. Nevertheless, one might wonder from what development stage is the L1 defect started to be evident in the het yotari brain? Was this mouse model analysed at embryonic stages? Could it be a problem in PP splitting at early development?

- References lacking during discussion, this should be revised (e.g. lines 465, 482).

- Lines 516-517: “understanding of human diseases caused by heterozygous loss-of-function in the *Dab1* gene”. Such as? This point could be further discussed.

## Author Response

General comments from reviewers:

The authors very carefully performed an extensive set of morphological examinations on cortical and hippocampal pyramidal neurons in the heterozygous yotari mouse, and revealed *Dab1* haploinsufficiency in their developmental behaviors. Cell visualization and tracking were nicely done, and the interpretations sound reasonable. 2

This study assesses the role of *Dab1* gene dosage in cerebral cortex development. DAB1 encodes for an adaptor protein known to be a key regulator of Reelin signaling in the control of neuronal migration and positioning in the developing brain. *Dab1* has been the subject of much research but the mechanisms mediating the reelin-Dab1 signaling pathway are yet not fully elucidated, including whether Reelin function depends on Dab1 dosage. The authors examined Dab1 haploinsufficiency by using the heterozygous yotari mouse model, which is an autosomal recessive null mutant of Dab1. Altogether, the authors were able to show convincingly that 1) Dab1 is haploinsufficient for maintaining the thickness of layer 1 and dendrite guidance of L2 pyramidal neurons in the cerebral cortex, although radial migration is not altered; 2) based on IHC and birthdating radial migration of late-born pyramidal neurons in the caudal hippocampus is sensitive to Dab1 dosage and heterozygous yotari display SP splitting, branching and misoriented dendrites.

This is an interesting and important question in the field and the experiments were well planned and executed.

The manuscript is well written and the images are of very good quality. Only minor corrections are required, mainly to help the readers understand the data and further discuss the results.

Response:

We are very encouraged that this reviewer found our work interesting and valuable.

Point-by-point responses to Reviewer’s comments

Point #1:

A brief discussion of whether the “subtle attenuation of the Reeling-Dab1 signaling pathway”, surely found in heterozygous yotari, could potentially be treated by REELIN exogenously administrated would be fruitful.

Response:

We thank the reviewer for this very interesting and important suggestions for the possible future clinical applications. We have discussed this possibility together with the next point #2.

Point #2:

It would be helpful to mention/discuss, in the introduction and/or discussion, the relevance and impact that this study could bring for the clinics. Which pathologies are described to be caused by Dab1 mutations, are they heterozygous or homozygous? The discussion of these literatures would strengthen the significance of this study.

Response:

We appreciate and agree with the reviewer. We have added new discussion about the clinical relevance of this study and pathology caused by homozygous Dab1 mutations. We have added the following text in the Introduction (lines 68-73): “and might be associated with the pathogenesis of a number of neuropsychiatric disorders including 3 schizophrenia (Impagnatiello et al., 1998; Guidotti et al., 2000), bipolar disorder (Guidotti et al., 2000), autism spectrum disorder (Fatemi et al., 2001; Fatemi et al., 2005), and Alzheimer’s disease (Chin et al., 2007). However, it is not clear whether there is Dab1 gene-dosage dependency for each reported phenotype.” We have also added the following text in the Discussion (line 586-line 609): “Similar to the Dab1 gene, the Reelin gene is also known to exhibit haploinsufficiency (Liu et al., 2001; Carboni et al., 2004; Qiu et al., 2006; Nullmeier et al., 2011; Bouamrane et al., 2016; Wang et al., 2019), and reduced Reelin levels have been observed in the brains of patients with schizophrenia (Impagnatiello et al., 1998; Guidotti et al., 2000), bipolar disorder (Guidotti et al., 2000), autism (Fatemi et al., 2001; Fatemi et al., 2005), and Alzheimer’s disease (Chin et al., 2007). Moreover, heterozygous reeler mice and Reelin ΔC-KI mice exhibit similar neurochemical, behavioral, and cognitive abnormalities to those observed in autism or schizophrenia (Costa et al., 2001; Tremolizzo et al., 2002; Carboni et al., 2004; Nullmeier et al., 2011; Sakai et al., 2016; Sanchez-Hidalgo et al., 2022). The anatomical abnormalities we observed in heterozygous yotari mice may also be related to behavioral defects because layer 1 and hippocampus are essential for higher-order cognitive functions (Hainmueller and Bartos, 2020; Schuman et al., 2021). Consistent with this possibility, behavioral abnormalities observed in schizophrenia, such as hyperactivity, decreased anxiety-like behavior, and impaired working memory, have been observed in dorsal forebrain-specific Dab1 conditional knockout mice (Imai et al., 2017). These findings suggest that therapeutic enhancement of Reelin signaling could prevent or ameliorate the cognitive deficits of schizophrenia and other neurodevelopmental disorders (Guidotti et al., 2016; Ishii et al., 2016; Hattori and Kohno, 2021). Indeed, administration of Reelin to mouse models of cognitive dysfunction such as Angelman syndrome and schizophrenia could prevent or ameliorate the cognitive deficits. (Hethorn et al., 2015; Ishii et al., 2015; Ibi et al., 2020; Sawahata et al., 2021). If a similar neuropsychiatric abnormality is observed due to Dab1 haploinsufficiency, it may be possible to ameliorate the defect by activating the Dab1 signaling through Reelin supplementation. “

Point #3:

- Line 46: missing Falconer 1951 reference.

Response:

Thank you for pointing this out. We have added the Falconer’s reference (line 43).

“Falconer DS (1951) Two new mutants, ‘trembler’ and ‘reeler’, with neurological actions in the house mouse (Mus musculus L.). J Genet 50:192-201.”

Point #4:

- line 52: ref Niu 2004 it’s a study in hippocampus, not in neocortex

Response: 4

We appreciate the reviewer for this comment. In accordance with the comment, we have changed the references (line 50) from: Niu S, Renfro A, Quattrocchi CC, Sheldon M, D’Arcangelo G (2004) Reelin promotes hippocampal dendrite development through the VLDLR/ApoER2-Dab1 pathway. Neuron 41:71-84. Olson EC, Kim S, Walsh CA (2006) Impaired neuronal positioning and dendritogenesis in the neocortex after cell-autonomous Dab1 suppression. Journal of Neuroscience 26:1767-1775. to Hamburgh M (1963) Analysis of the Postnatal Developmental Effects of “Reeler,” a Neurological Mutation in Mice. A Study in Developmental Genetics. Dev Biol 8:165-185. Caviness VS, Jr. (1982) Neocortical histogenesis in normal and reeler mice: a developmental study based upon [3H]thymidine autoradiography. Brain Res 256:293-302. D’Arcangelo G (2005) The reeler mouse: anatomy of a mutant. Int Rev Neurobiol 71:383-417.

Point #5:

- lines 64/65: don’t understand the meaning of the sentence “Various studies of haploinsufficiency suggested that genes which show haploinsufficiency have some tendency for its functions.”

Response:

We thank the reviewer for the comment. We have changed the following text in the Results section (lines 58- 61) from: “Various studies of haploinsufficiency suggested that genes which show haploinsufficiency have some tendency for its functions. Genes encoding enzymes are relatively dose-insensitive, while genes encoding proteins involving structural, transcriptional, or regulatory functions are more likely to be dose-sensitive (Kondrashov and Koonin, 2004; Morrill and Amon, 2019).” to “In general, genes encoding enzymes tend to be dose-insensitive, whereas genes that encode proteins with structural, transcriptional, or regulatory functions are more likely to be dose-sensitive (Kondrashov and Koonin, 2004; Morrill and Amon, 2019).”

Point #65

- Line 87 authors say “than in other brain regions”. Which other brain regions are these? Were they analysed in this study?

Response

Thank you for pointing this out. We agree with the reviewer that our description was unclear. We have changed the following text in the Introduction (lines 82-85) from: “These findings suggest that the positioning and dendrite guidance of superficial-layer neurons in the cerebral neocortex and the migration of late-born pyramidal neurons in the caudal hippocampus have a greater dependence on Dab1 protein amount than in other brain regions.” to “These findings suggest that the Dab1 gene dosage is important for maintenance of the layer 1 thickness and dendrite guidance of superficial-layer neurons in the cerebral neocortex and for the migration of late-born pyramidal neurons in the caudal hippocampus.”

Point #7

- Figure 1: authors describe comparisons in neocortex and hippocampal structures at P7 at 2 brain levels, one caudal at the level of visual cortex, and another rostral at the level of motor cortex (line 319). However, the images in Fig.1C-D do not represent motor cortex, which should be much more rostral. The close-ups C’ and D’ are in fact S1-S2 cortex. Similarly, the authors mention visual cortex for the caudal level, but the close-ups A’ and B’ represent auditory cortex. Even if the authors wanted to mention visual cortex just as a reference for the selected caudal section, why not mentioning auditory cortex since that is what is shown in the close-ups?

Response

We thank and agree with the reviewer for this comment. The description of the brain region we observed was unclear. In accordance with the comment, we have changed the following text in the Result (line 322-line 325) from: “we found that heterozygous yotari mutant mice at P7 had a thinner layer 1 in the cerebral neocortex compared with that of wild-type mice both at caudal (visual) cortex and rostral (motor) cortex (Fig. 1).” to “but layer 1 was significantly thinner in both the rostral brain region containing somatosensory and auditory cortices (Fig. 1, Rostral) and caudal brain region containing visual and auditory cortices (Fig.1 Caudal) of P7 heterozygous yotari mutant mice compared to P7 wild-type mice.” 6

We have also added an illustration to Figure 1 to clearly show the regions we analyzed and changed the following text in the Figure legends (lines 914-921) from: “Caudal (A-B’) or rostral (C-D’) region of wild-type (+/+; A, A’, A”, C, C’, C”) or heterozygous (+/yot; B, B’, B’, D, D’, D”) cerebral neocortex and hippocampus at P7 were analyzed using Nissl staining. Enlarged views from the regions enclosed by rectangles in A, B, C, and D are shown in A’, A’, B’, B’, C’, C’, D’, D’, respectively.” to “(A) Schematic illustrations of the analyzed brain regions in B-E’. S: somatosensory cortex; A: auditory cortex; V: visual cortex. (B-E’) Nissl-stained coronal brain sections of the caudal region containing the visual and auditory cortices (B-C’, Caudal) and rostral region containing the somatosensory and auditory cortices (D-E’, Rostral) from wild-type mice (+/+; B, B’, B”, D, D’, D”) and heterozygous yotari mice (+/yot; C, C’, C’, E, E’, E”) at P7. Enlarged views from the regions enclosed by rectangles in B, C, D, and E are shown in B’, B’, C’, C’, D’, D’, E’, E’, respectively. B’ and C’ show auditory cortex, and D’ and E’ show somatosensory cortex.”

Point #8

- Figure 2: Same comment as before concerning the brain areas represented in images A-F according to the description in the figure legend. Is A-B motor cortex, based on the images sections chosen in figure 1C-D?

The authors say that 3 different sections were obtained: caudal (visual), middle (auditory), rostral (motor) cortex, so sections mean “brain regions”? I would consider sections as slice sections in the rostrocaudal axis, and the visual and auditory cortex would be in the same caudal section. I think this part can be a little confusing for the reader to understand which sections/brain areas were used in each analysis. These descriptions should be rethought.

Response

We appreciate the reviewer for this comment. In accordance with the comment, we have changed the following text in the Result (lines 326-336) from: “To quantitatively evaluate the layer 1 thickness, we stained the brain sections with DAPI (Fig. 2A-F) and measured the layer 1 thickness (Fig. 2H), cortical wall thickness (Fig. 2J), and then calculated the relative layer 1 thickness (Fig. 2I).” to “To quantitatively evaluate potential differences in neocortical layer thickness between wild-types and heterozygous yotari mutant mice, we stained nuclei in coronal brain sections with DAPI (Fig. 2B-G, 2B’- G’) and measured layer 1 thickness (Fig. 2I), cortical wall thickness (Fig. 2K) (see Methods). Consistent with Nissl staining, layer 1 thickness (Fig. 2I) of the rostral brain region containing somatosensory and 7 auditory cortices (Fig. 2 and Fig. 2-1, Rostral), middle brain region containing visual, parietal, and auditory cortices (Fig. 2 and Fig. 2-1, Middle), and caudal brain region containing visual and auditory cortices (Fig. 2 and Fig. 2-1, Caudal) were significantly reduced in yotari heterozygous mice compared to wild-type mice. Moreover, relative layer 1 thickness, calculated as the ratio of layer 1 thickness to cortical wall thickness, was also reduced in rostral, middle, and caudal regions of heterozygous yotari mouse neocortex (Fig. 2J).” We have also added an illustration to Figure 2 to clearly show the regions we analyzed and changed the following text in the Figure legend (line 927-line 932) from: “(A-F’) DAPI staining of wild-type (+/+; A, A’, C, C’, E, E’) or heterozygous (+/yot, B, B’, D, D’, F, F’) mouse cerebral neocortex at P7 from three different parts in rostral-caudal axis.” to “(A) Schematic illustrations of the analyzed brain regions in B-E’. S: somatosensory cortex; A: auditory cortex; V: visual cortex. (B-E’) Nissl-stained coronal brain sections of the caudal region containing the visual and auditory cortices (B-C’, Caudal) and rostral region containing the somatosensory and auditory cortices (D-E’, Rostral) from wild-type mice (+/+; B, B’, B”, D, D’, D”) and heterozygous yotari mice (+/yot; C, C’, C’, E, E’, E”) at P7. Enlarged views from the regions enclosed by rectangles in B, C, D, and E are shown in B’, B’, C’, C’, D’, D’, E’, E’, respectively. B’ and C’ show auditory cortex, and D’ and E’ show somatosensory cortex.”

Point #9

Moreover, in the Materials and Methods line 242, it’s mentioned that the analysis was done in a region of SS cortex but the figure legend indicates that 11 positions were measured per section (visual, auditory, motor). So, I couldn’t really understand what region(s) the data represent.

Response

We appreciate and agree with the reviewer for this comment. In accordance with the comment, we clarified this point by adding new Extended Fig. 2-1 and modified the text in the Result (lines 326-336) from: “To quantitatively evaluate the layer 1 thickness, we stained the brain sections with DAPI (Fig. 2A-F) and measured the layer 1 thickness (Fig. 2H), cortical wall thickness (Fig. 2J), and then calculated the relative layer 1 thickness (Fig. 2I). As a result, the layer 1 thickness was thinner in yotari heterozygous mice than that in wild-type mice (Fig. 2H).” to “To quantitatively evaluate potential differences in neocortical layer thickness between wild-types and heterozygous yotari mutant mice, we stained nuclei in coronal brain sections with DAPI (Fig. 2B-G, 2B’- 8 G’) and measured layer 1 thickness (Fig. 2I), cortical wall thickness (Fig. 2K) (see Methods). Consistent with Nissl staining, layer 1 thickness (Fig. 2I) of the rostral brain region containing somatosensory and auditory cortices (Fig. 2 and Fig. 2-1, Rostral), middle brain region containing visual, parietal, and auditory cortices (Fig. 2 and Fig. 2-1, Middle), and caudal brain region containing visual and auditory cortices (Fig. 2 and Fig. 2-1, Caudal) were significantly reduced in yotari heterozygous mice compared to wild-type mice. Moreover, relative layer 1 thickness, calculated as the ratio of layer 1 thickness to cortical wall thickness, was also reduced in rostral, middle, and caudal regions of heterozygous yotari mouse neocortex (Fig. 2J).”

We have also changed the text in Figure 2 legend (line 927-line 932) from: “(A-F’) DAPI staining of wild-type (+/+; A, A’, C, C’, E, E’) or heterozygous (+/yot, B, B’, D, D’, F, F’) mouse cerebral neocortex at P7 from three different parts in rostral-caudal axis.” to “(A) Schematic illustrations showing brain regions analyzed for layer 1 and cortical wall thickness. S: somatosensory cortex; A: auditory cortex; V: visual cortex, P: parietal cortex. (B-G’) DAPI staining of wild-type (+/+; B, B’, D, D’, F, F’) and heterozygous yotari (+/yot, C, C’, E, E’, G, G’) mouse cerebral neocortex at P7. Representative images obtained from the somatosensory (Rostral: B, B’, C, C’), parietal (Middle: D, D’, E, E’), and visual (Caudal: F, F’, G, G’) cortex.” , and from (line 936): “See Materials and Methods for details.” to “See Materials and Methods and Fig. 2-1 for details.” We have newly added the following text in the Figure legend (line 1081-line 1089). “Figure 2-1. Schematic illustrations showing the three brain regions along the rostro-caudal axis and the 11 positions analyzed in each brain region.

Schematic illustrations of the three brain regions analyzed for layer 1 and cortical wall thickness, the rostral region containing the somatosensory and auditory cortices (Rostral), middle brain region containing the visual, parietal, and auditory cortices (Middle), and the caudal brain region containing the visual and auditory cortices (Caudal). In the lower panel, the 11 positions analyzed in each brain region (A, A+1-5, A-1-5) are shown by dashed lines. S: somatosensory cortex; A: auditory cortex; V: visual cortex, P: parietal cortex.” 9

Point #10

The authors show a clear reduction in L1 thickness in yotari mice (fig2H) but the relative thickness is ns, although almost, which is surprising since the cortical wall thickness is not different. Maybe some regions present more differences in thickness than others and by pooling all data these differences might be lost. Showing this analysis separated by brain region could be insightful.

Response

We appreciate the reviewer for this important suggestion. In accordance with the suggestion, we have reanalyzed layer 1 and cortical wall thickness at three different regions, and added newly analyzed data. We have changed Fig. 2 and changed the text in the Results (lines 329-336) from: “As a result, the layer 1 thickness was thinner in yotari heterozygous mice than that in wild-type mice (Fig. 2H).” to “Consistent with Nissl staining, layer 1 thickness (Fig. 2I) of the rostral brain region containing somatosensory and auditory cortices (Fig. 2 and Fig. 2-1, Rostral), middle brain region containing visual, parietal, and auditory cortices (Fig. 2 and Fig. 2-1, Middle), and caudal brain region containing visual and auditory cortices (Fig. 2 and Fig. 2-1, Caudal) were significantly reduced in yotari heterozygous mice compared to wild-type mice. Moreover, relative layer 1 thickness, calculated as the ratio of layer 1 thickness to cortical wall thickness, was also reduced in rostral, middle, and caudal regions of heterozygous yotari mouse neocortex (Fig. 2J).”

We have also changed the text in Figure 2 legend (lines 941-945) from: “Three different sections were obtained from caudal (visual) cortex, middle (auditory), and rostral (motor) cortex, 11 positions were measured per each section and total 33 positions were measured for each brain. After calculation of the mean values from each brain, statistical significance was analyzed by unpaired Student’s t-test and the p-values are shown on the graph.” to “Measurement was performed on 6 brains obtained from 6 different litters (n = 6). Three different coronal sections were obtained from the caudal, middle, and rostral brain of each mouse as shown in A, and 11 positions were analyzed per brain region. Mean values were then calculated for each brain region. Genotype differences were analyzed by Student’s t-test.”

Point #11

10

- Figure 3 author say: “we found that Brn2-positive layer 2/3 neurons in yotari heterozygous mice tended to locate more distantly from the VS than those in wt”. Authors assess their position using BrdU birthdating assays showing no changes in the pattern and position of these neurons. However, it’s true that in graph D and in images E, L2/3 looks more extended and cells more disperse, as if L2/3 was pushed up because L1 is thinner. Is this observed at all brain levels analysed? And about cell number/density? Are the number of Brn2+ or BrdU+ alone the same? And number of BrdU+/Brn2+? Could the thickness of individual layers be altered?

Response

We appreciate the reviewer for this comment. In accordance with the comment, we carried out (1) positioning analysis of the TBR1-, RORB-, or BRN2-positive neurons at three different brain regions along the rostro-caudal axis (Fig. 3), (2) cell number and cell density analysis of DAPI-positive cells (Extended Fig. 3-1), (3) cell number and cell density analysis of BRN2 single positive, BrdU single positive, or BRN2/BrdU double positive cells (Extended Fig. 4-1), (4) positioning analysis of the BRN2/BrdU double positive cells at three different brain regions along the rostro-caudal axis (Fig. 4), and (5) analysis of individual layer thickness (Extended Fig. 3-2). As the result of these experiments, we found that half reduction of Dab1 gene dosage causes a decrease in the layer 1 thickness as well as an increase in the area of the cortical region and cell number with an expansion of the RORB-positive layer 4 thickness. We have split the original Fig. 3 into new Fig. 3 and new Fig. 4, and added data. We have also added new figure as Extended Fig. 3-1, 3-2, and 4-1. In accordance with the changes and additions to the Figures, we have changed and added the text in the Materials and Methods, Results, and figure legends as follows. We have added the following text in the Materials and Methods section (lines 268-276). “Measurement of neocortical layer thickness by the distribution of layer marker-positive cells For quantitative comparisons, we defined layer thickness as the distance between the 20th and 80th percentile of layer marker-positive cells (Fig. 3-2A). The markers used were TBR1, RORB, or BRN2.

Measurement was performed using coronal sections of somatosensory, parietal, and visual cortices obtained from three brains from different litters. Then the average thickness of each brain along the rostro-caudal axis was calculated. Results were compared between groups by independent samples Student’s t-test using the R language environment.”

We have changed the subsection title in the Results section (lines 341-342) from: “Heterozygous yotari cerebral neocortex does not have significant changes in the position of cells positive for TBR1, RORB, or BRN2” to “The RORB-positive layer 4 but not TBR1- and BRN2-positive layers 5/6 and 2 are expanded in the 11 neocortex of heterozygous yotari mice” We have also changed the text in the Result section (lines 351-377) from: “However, when we observe the distribution of the raw data shown by the scatter plot, we found that BRN2-positive layer 2/3 neurons in yotari heterozygous mice tended to locate more distantly from the ventricular surface than those in wild-type mice (Fig. 3D).” to “While there were no significant differences in relative position of cells (Fig. 3C), scatter plots revealed that BRN2-positive layer 2/3 neurons tended to be located farther from the ventricular surface in heterozygous yotari mice than wild-type mice (Fig. 3D), suggesting that layer 2/3 is shifted by cell dispersion into layer 1.

We then examined if this reduction in layer 1 thickness was accompanied by changes in other layers. Briefly, we defined a 200-m wide measurement area from the layer 1-2 boundary to the ventricular surface (Fig. 3-1A) and examined layer 2/3, 4, and 5/6 area (Fig. 3-1B), cell number (Fig. 3-1C), and cell density (Fig. 3-1D) in somatosensory (Rostral), parietal (Middle), and visual (Caudal) cortices. We found that the measured area was significantly larger in the middle and caudal brain regions of heterozygous yotari mice (Fig. 3-1B). There was also a significant increase in cell number within the middle brain region of heterozygous yotari mice, and a numerical increase in both rostral and caudal brain regions that did not reach statistical significance (Fig. 3-1C). In contrast, there were no differences in cell density between wild-type and heterozygous yotari mutant mice (Fig. 3-1D).

We also examined the thickness of each layer by measuring the distributions of neurons immunopositive for layer-specific markers (Fig. 3-2). For comparison, we defined the layer thickness as the length along which the 20th-80th percentile population of layer marker-positive cells is distributed (Fig. 3-2A) as it was difficult to clearly determine the boundaries of layers based on the distribution of the all layer marker positive cells. This analysis revealed no significant difference in TBR1-positive layer 5/6 thickness or BRN2-positive layer 2/3 thickness, but RORB-positive layer 4 thickness was greater in heterozygous yotari mice than wild-type mice (Fig. 3-2B). These results suggest that a half reduction in the Dab1 gene dosage causes a decrease in layer 1 thickness as well as an increase in the area of the neocortical region from layer 2 to the ventricular surface and cell number with an expansion of RORB-positive layer 4 thickness.”

We have added the following text in the Results section (lines 384-387). “Similarly, there were no significant differences in the number of E16.5-BrdU-labeled cells, BRN2-positive cells, and BrdU/BRN2 double-positive cells in a 200-μm wide region from the layers 1-2 boundary to the ventricular surface between wild-type and heterozygous yotari mice (Fig. 4-1).”

We have added the following text in the Discussion section (lines 570-574). 12 “It is also possible that the increase in cell number within other neocortical layers (Fig. 3-1) leads to a reduction in layer 1 thickness. The causal relationship between the increase in cell number and the expansion of the neocortical area, and why the cell number increased, are important subjects for future study.”

We have changed the following text in Figure legend of Fig. 3 (lines 950-953) from: “(A) Coronal neocortical sections obtained from P7 wild-type (+/+) or heterozygous yotari mice (+/yot) were stained for TBR1, RORB, or BRN2.” to “(A) TBR1-, RORB-, or BRN2-positive cells in the somatosensory (Rostral), parietal (Middle), and visual (Caudal) cortices of P7 wild-type (+/+) and heterozygous yotari mice (+/yot). Representative images of coronal neocortical sections in the middle brain region are shown.”

We have also changed the following text in the Figure legend of Fig. 3 (lines 956-959) from: “(C and D) Combination of dot, violin, and scatter plots show relative position of layer marker-positive cells (C) and distance of layer marker-positive cells from the ventricular surface (D).” to “(C and D) Combination of dot, violin, and scatter plots showing the relative positions of layer marker-positive cells (C) and distances of layer marker-positive cells from the ventricular surface (D) within three brain regions along the rostro-caudal axis.”

We have additionally changed the following text in Figure legend of Fig. 3 (lines 963-967) from: “After calculation of the mean values from each brain, statistical significance was analyzed by Mann-Whitney U test (F) or unpaired Student’s t-test (G)” to “After calculation of mean values for each brain, most group differences were analyzed by independent samples Student’s t-test; however, some experiments were analyzed by Mann-Whitney U-test (C, BRN2 (Middle)) or unpaired Welch’s t-test (C, TBR1 (Rostral), D, TBR1 (Rostral)).”

As we have split the original Fig. 3 into new Fig. 3 and new Fig. 4, we have added the title of figure as follows (lines 970-971). 13 “Figure 4. There were no significant positional changes in late-born, BRN2-positive, superficial neocortical neurons in yotari heterozygous mice.”

As we have newly added Extended Fig. 3-1, 3-2, 4-1, we have added their legends as follows (lines 1091- 1138). “Figure 3-1. The neocortical region from layer 2 to the ventricular surface of heterozygous yotari mice is expanded and the number of cells is increased but there is no change in the cell density. (A) Schematic showing the dorsolateral cerebral neocortex. The measurement area was from the boundary of layer 1 (L1) and layer 2 (L2) to the ventricular surface (VS) (200 μm width, dark gray area). PS: pial surface; L6: layer 6. (B-D) Sections of the rostral brain region containing the somatosensory and auditory cortex (Rostral), middle brain region containing the visual, parietal, and auditory cortex (Middle), and caudal brain region containing the visual and auditory cortex (Caudal) from 6 wild-type (W) and heterozygous yotari mice (He) each, all from different litters. Confocal images were then taken of the somatosensory, parietal, and visual cortex, respectively. Numbers of DAPI-positive cells within the measurement region are shown in (C) and cell density in (D) on combination dot and scatter plots. The black dots with a black bar indicate the mean and standard deviation, respectively, and each gray dot is a raw datapoint. The total numbers of analyzed mice are shown below each graph. Statistical significance was evaluated by independent samples Student’s t-test (B, C, D: Middle) or Mann-Whitney U-test (D: Rostral and Caudal). The p-values are labeled on the graph. *p < 0.05, ** p < 0.01, ns (not significant) p > 0.05. Figure 3-2. Heterozygous yotari mice exhibit an expanded RORB-positive cell layer compared to wild-type mice. (A) Schematic illustration showing how layer thickness was defined as the distance from the 20th to 80th percentile of layer marker-positive cells. (B) Thicknesses of TBR1-, RORB-, or BRN2-positive cell layers measured on coronal sections of the somatosensory (Rostral), parietal (Middle), and visual (Caudal) cortex from three wild-type (W) and three heterozygous yotari mice (He), each of different litters. The black dot and bar in the leftmost dot plot indicate the mean and standard deviation, respectively, each gray dot indicates the mean from one brain, and each black dot in the scatter plot is a datapoint obtained from the rostral, middle, or caudal brain regions. The total number of analyzed coronal brain sections is shown at the bottom of the graph. Groups were compared by independent samples Student’s t-test. The p-values are labeled on the graph. **p < 0.01, ns (not significant) p > 0.05. Figure 4-1. There were no significant changes in number and cell density of E16.5-BrdU-labeled cells, BRN2-positive cells, and BrdU/BRN2 double-positive cells between wild-type and heterozygous yotari mice.

The numbers of BrdU/BRN2 double-positive cells (A), E16.5-BrdU-labeled cells (C), and BRN2-positive cells (E) were counted in a 200-μm wide region between the layer 1-2 boundary and ventricular surface (shown in Figure 3-1A). Corresponding cell densities were then calculated (B, D, F). Measurements were performed in the somatosensory (rostral), parietal (middle), and the visual (caudal) cortex of three wild-type 14 (W) and three yotari heterozygous mice (He), each from different litters. The large black dots and black bars in the graph represent the mean and standard deviation, respectively. The gray dots indicate the mean from one brain and each small black dot in the scatter plot is a raw datapoint obtained from the rostral, middle, or caudal brain region. The total number of analyzed brain sections is shown at the bottom of the graph. Groups were compared by Mann-Whitney U-test (A) or independent samples Student’s t-test (B-F). The p-values are labeled on the graph. ns (not significant) p > 0.05.”

Point #12

Brain section/level for images in Fig.3A should be mentioned in results and figure legend.

Response

We thank the reviewer for this comment. We have changed the following text in Figure legend of Fig. 3A (line 950-953) from: “(A) Coronal neocortical sections obtained from P7 wild-type (+/+) or heterozygous yotari mice (+/yot) were stained for TBR1, RORB, or BRN2.” to “(A) TBR1-, RORB-, or BRN2-positive cells in the somatosensory (Rostral), parietal (Middle), and visual (Caudal) cortices of P7 wild-type (+/+) and heterozygous yotari mice (+/yot). Representative images of coronal neocortical sections in the middle brain region are shown.”

Point #13

- Figure 4: what about the percentage of cells in the pyr layer, is it changed in the mutant? Was this analysed?

Response

We thank the reviewer for this comment. The percentage of BrdU+/CTIP2+ cells in the SO region was calculated by dividing the number of cells distributed in the SO region by the combined number of cells distributed in the SO and Pyr regions. Therefore, the distribution ratio in the Pyr region is also analyzed. In order to show the distribution ratio in both regions, new Fig. 5 (the number of figure has been changed from 4 to 5) has been modified to show the ratio of distribution in the SO region and the Pyr region.

Point #14

Substitute “BCL11B” in lines 363 and 810. Bcl11b is the name of the gene, Ctip2 is the protein.

Response

We thank the reviewer for this comment. We have substituted the BCL11B to CTIP2. In addition, we have made the corresponding changes from BCL11B to CTIP2 in other parts of the text and in the Figure 5. 15

Point #15

- Figure 5: wrong figure reference in line 406.

Response

We thank the reviewer for this comment. We have corrected the figure reference number.

Point #16 Are the mispositioned neurons in SO still migrating neurons? The horizontal orientation of the cell soma seems to indicate that.

Response We thank and agree with the reviewers’ comment. We have added the description of the possibility in the discussion as follows (lines 579-582). “it is also possible that these abnormalities are secondary to migration failure or that neurons ectopically distributed in the SO region are still migrating and these misoriented structures are leading processes rather than dendrites.”

Point #17

Authors describe a tendency in yotari mice for having shorter total length of apical dendrites because neurons in both pyr and SO layers were analysed. Neurons in SO look either immature or migrating still, but looking at neurons is pyr layer, is this tendency still observed? They look very similar when comparing images E with images B-C.

Response

We thank the reviewer for this comment. We have additionally measured the morphology of the neurons within the pyramidal cell layer in the yotari heterozygous mice and performed statistical analysis among neurons distributed in the pyramidal cell layer in wild-type brains, neurons ectopically distributed in the SO region and those normally distributed in the pyramidal cell layer in yotari heterozygous mouse brains. Because of the change of the statistical methods for analysis among the three groups, the previously observed significant differences between neurons from wild-type mice and ectopically positioned yotari heterozygous mice were not observed for angle of apical dendrites and total number of branching points, while a significant difference was observed for the total length of apical dendrites. We also observed a decreasing tendency of the total length of apical dendrites and the total number of branching points in the neurons distributed in the pyramidal cell layer in the yotari heterozygous mice compared to the wild-type neurons, respectively. We have added the data of neurons normally distributed in the pyramidal cell layer in yotari heterozygous mouse brains to Fig. 6F-H, and changed the text in the Results section (lines 434-448) from: “To compare the cellular morphology, mCherry-positive cells distributed within the pyramidal cell layer in the wild-type mice (Fig. 5 B-C’) and mCherry-positive cells ectopically distributed within the SO in the heterozygous 16 yotari mice (Fig. 5 D-E’, arrowheads) were observed, respectively. The total length, total number of branching points, and angle of the primary apical dendrite were analyzed (Fig. 5F-H). We found that there was a significant reduction of the total number of branching points in the heterozygous yotari mice (Fig. 6G).

Furthermore, statistically significant number of neurons in heterozygous yotari mice had misoriented primary apical dendrites (Fig. 5H). Although no statistical difference was observed in the total length of apical dendrites, many of the neurons in the heterozygous yotari mice tended to have relatively shorter total length of apical dendrites than in the wild type mice (Fig. 5F).” to “We then compared apical dendrite morphology among (1) mCherry-positive cells distributed within the pyramidal cell layer of wild-type mice (Fig. 6B-C’), (2) mCherry-positive cells normally distributed within the pyramidal cell layer of heterozygous yotari mice (Fig. 6E and E’, yellow arrowheads), and (3) mCherry-positive cells ectopically distributed within the SO of heterozygous yotari mice (Fig. 6D-E’, cyan arrowheads). Total apical dendrite length was significantly shorter among ectopically positioned neurons in heterozygous yotari mice compared to wild-types (Fig. 6F). Total apical dendritic length was also numerically reduced among normally positioned neurons in heterozygous yotari mice compared to wild types, although the difference did not reach statistical significance. In addition, the total number of branching points was numerically reduced among normally positioned and ectopically positioned neurons of heterozygous yotari mice compared to wild-types (Fig. 6G).

Furthermore, many of the primary apical dendrites in ectopically positioned neurons of heterozygous yotari mice were orientated in abnormal directions, although the average did not differ significantly between genotypes (Fig. 6H).” We also changed the text in the figure legend (lines 1025-1044) from: “Representative images of the mCherry-positive cells in the pyramidal cell layer (pyr) in wild-type mice (B-C’) or ectopically distributed cells indicated by arrowheads in the SO in yotari heterozygous mice (D∼E’) are shown. The ectopic cell regions in SO in yotari heterozygous mice are indicated by double-headed arrows in SO (D and E). (F-H) Measurement data of the total length of apical dendrites (F), the total number of branching points of apical dendrites (G), and the angle of apical dendrites (H) obtained from 3 different wild-type (+/+) or 6 different yotari heterozygous mice (+/yot) from different litters are plotted by combination of dot, violin, and scatter plots. Black dot and the bar in the leftmost dot plot indicate mean and the standard deviation, respectively, and each gray dot indicates mean of the data from 1 brain. Each black circle in the scatter plot indicates raw data. Total analyzed cell numbers are shown below each graph. After calculation of the mean values from each brain, statistical significance was calculated by unpaired Welch’s t-test (F and H) or unpaired Student’s t-test (G), and the p-values are labeled on the graph.” to “Representative images of mCherry-positive cells in the pyramidal cell layer (pyr) of wild-type mice (B-C’), 17 normally distributed cells in the pyramidal cell layer of heterozygous yotari mice (yellow arrowheads in E and E’), and ectopically distributed cells in the SO (cyan arrowheads in D-E’) of heterozygous yotari mice (D-E’). The ectopic cell regions in the SO of heterozygous yotari mice are indicated by double-headed arrows (D and E). (F-H) Total length of apical dendrites (F), total number of branching points (G), and angle of apical dendrites (H) obtained for 26 neurons from 3 wild-type (WT) mice, 8 normally distributed neurons from 3 heterozygous yotari mice (He, normal), and 14 ectopically distributed neurons from 6 heterozygous yotari mice (He, ectopic) are plotted by a combination of dot and scatter plots. All analyzed mice were obtained from different litters. The large black dot and the bar in the leftmost dot plot indicate the mean and standard deviation, respectively, and the gray dots indicate the mean from one brain. Each small black dot in the scatter plot is a raw datapoint. The total number of analyzed cells is shown below each graph. After calculation of the mean values from each brain, groups were compared by Tukey-Kramer test (F and G) or Steel-Dwass test (H). The p-values are labeled on the graph. *p < 0.05, ns (not significant) p > 0.05. The schematic drawing in H shows a pyramidal neuron in the pyramidal cell layer (pyr). The angle (θ) of the primary apical dendrite was determined as the angle between a perpendicular line passing through the center of the neuron and the first branch point.”

Point #18

- Figure 6: in panel B-C, it looks like the cortex in +/yot is bigger than WT and it was shown no differences in the cortical wall length (Fig.2J), so this representation can be a little misleading to the reader.

Response

We thank the reviewer. We have changed the image of the +/+ cortex to more appropriate one so that the uppermost portion of the image is aligned with the pial surface.

Point #19

Authors say in line 429-430 “in the heterozygous yotari mice the apical dendrites of layer 2 neurons do not appear to be directed in the layer 1 (Fig. 6C)”. It’s too of a strong affirmation, since is still possible to observe apical dendrites in L1. The frequency of sprouting into L1 was not analysed.

Response

We thank the reviewer for pointing this out. We agree with this opinion. We have deleted the following text from the result section. “When we introduced the plasmids into the wild-type mouse brain, we observed that many apical dendrites of layer 2 neurons were directed toward the brain surface and invaded the layer 1 (Fig. 7B). However, in the heterozygous yotari mice the apical dendrites of layer 2 neurons do not appear to be directed into the layer 1 (Fig. 7C).”

Point #20

- Line 487: authors hypothesize that reduction of L1 in het yotari might be caused after neurons finish their 18 radial migration and due to the abnormalities in their apical dendrites that probably do not sustain the further development of L1. This is a very interesting point, that is supported by many other mouse models with similar phenotypes, and which the authors discuss extensively. Nevertheless, one might wonder from what development stage is the L1 defect started to be evident in the het yotari brain? Was this mouse model analysed at embryonic stages? Could it be a problem in PP splitting at early development?

Response We thank the reviewer for this important comment. We do not have the data on whether the layer 1 defects in the yotari heterozygous mice are also observed at earlier stages of development, including when preplate splitting occurs. When we observed the structure of the neocortex by Nissl staining at P0, there seemed to be no significant difference between wild-type and heterozygous mice. Therefore, it seems reasonable to assume that reduction of layer 1 thickness occurs after P0. Although we do not have the clear answer to respond to the question at present, we also think it is important to clarify this point in the future. We have added Nissl staining data at P0 as Extended Fig. 1-1. We also added the figure legend as follows (lines 1077-1079). “Figure 1-1. Nissl staining of the cerebral cortex at P0. Nissl-stained coronal sections of cerebral cortex from wild-type (+/+) and heterozygous yotari (+/yot) mice obtained at P0. Scale bar: 1 mm.”

Point #21

- References lacking during discussion, this should be revised (e.g. lines 465, 482).

Response

We thank the reviewer for this comment. We have cited the following references at the end of “Homozygous null mutation of the Dab1 gene causes severe migration failure both in the cerebral neocortex and hippocampus “ (lines 491-493).

Howell BW, Hawkes R, Soriano P, Cooper JA (1997) Neuronal position in the developing brain is regulated by mouse disabled-1. Nature 389:733-737. Yoneshima H, Nagata E, Matsumoto M, Yamada M, Nakajima K, Miyata T, Ogawa M, Mikoshiba K (1997) A novel neurological mutant mouse, yotari, which exhibits reeler-like phenotype but expresses CR-50 antigen/reelin. Neurosci Res 29:217-223. Franco SJ, Martinez-Garay I, Gil-Sanz C, Harkins-Perry SR, Muller U (2011) Reelin regulates cadherin function via Dab1/Rap1 to control neuronal migration and lamination in the neocortex. Neuron 69:482-497. Blume M, Inoguchi F, Sugiyama T, Owada Y, Osumi N, Aimi Y, Taki K, Katsuyama Y (2017) Dab1 contributes differently to the morphogenesis of the hippocampal subdivisions. Dev Growth Differ 59:657-673. 19

We have added the following references at the end of “Homozygous null mutation of the Dab1 gene also causes complete loss of cerebral neocortex layer 1” (lines 508-510). Sweet HO, Bronson RT, Johnson KR, Cook SA, Davisson MT (1996) Scrambler, a new neurological mutation of the mouse with abnormalities of neuronal migration. Mamm Genome 7:798-802. Howell BW, Hawkes R, Soriano P, Cooper JA (1997) Neuronal position in the developing brain is regulated by mouse disabled-1. Nature 389:733-737. Ware ML, Fox JW, Gonzalez JL, Davis NM, Lambert de Rouvroit C, Russo CJ, Chua SC, Jr., Goffinet AM, Walsh CA (1997) Aberrant splicing of a mouse disabled homolog, mdab1, in the scrambler mouse. Neuron 19:239-249.

Yoneshima H, Nagata E, Matsumoto M, Yamada M, Nakajima K, Miyata T, Ogawa M, Mikoshiba K (1997) A novel neurological mutant mouse, yotari, which exhibits reeler-like phenotype but expresses CR-50 antigen/reelin. Neurosci Res 29:217-223.

Point #22

- Lines 516-517: “understanding of human diseases caused by heterozygous loss-of-function in the Dab1 gene”. Such as? This point could be further discussed.

Response

We thank the reviewer for this comment. As this study is the first report showing that Dab1 gene has a haploinsufficiency for the structure of the cerebral cortex, it is not yet known whether there is a similar Dab1 gene haploinsufficiency for those structure in humans. Thus, we have added the following text in the Discussion (lines 534-539), and as to the relevance to human disease we have added the description in another part of the Discussion, as described above. “While it is yet unknown if haploinsufficiency of the Dab1 gene has similar effects on human cortical and hippocampal development, haploinsufficiency of the related Reelin gene is associated with various neuropsychiatric disorders (Guidotti et al., 2016; Ishii et al., 2016; Hattori and Kohno, 2021). Given the critical role of Dab1 in the canonical Reelin signaling pathway (Honda et al., 2011; Jossin, 2020), the findings of this study could provide potential explanations for human diseases.” Other points that we have changed.

We have changed the title of this manuscript from: “Heterozygous Dab1 null mutation causes defect in neocortical and hippocampal development” 20 to “Heterozygous Dab1 null mutation disrupts neocortical and hippocampal development”

We have also changed the abbreviated title from: “Haploinsufficiency of Dab1 gene” to “Dab1 haploinsufficiency disrupts neurodevelopment”
